# *Colletotrichum* species associated with mango in southern China

**DOI:** 10.1038/s41598-019-54809-4

**Published:** 2019-12-11

**Authors:** Qili Li, Junyan Bu, Juan Shu, Zhihe Yu, Lihua Tang, Suiping Huang, Tangxun Guo, Jianyou Mo, Shuming Luo, Ghulam Sarwar Solangi, Tom Hsiang

**Affiliations:** 1 Institute of Plant Protection, Guangxi Academy of Agricultural Sciences and Guangxi Key Laboratory of Biology for Crop Diseases and Insect Pests, Nanning, Guangxi 530007 China; 2 College of Life Sciences, Yangtze University, Jingzhou, Hubei 434025 China; 3 Plant Breeding Institute, The University of Sydney, Cobbitty, NSW 2570 Australia; 4 Department of Entomology, Sindh Agriculture University Sub-campus, Umerkot, 69100 Pakistan; 5 Environmental Sciences, University of Guelph, Guelph, Ontario Canada

**Keywords:** Phylogenetics, Fungal biology

## Abstract

Mango (*Mangifera indica* L.) is an economically significant fruit crop in provinces of southern China including Hainan, Yunnan, Sichuan, Guizhou, Guangdong and Fujian. The objective of this study was to examine the diversity of *Colletotrichum* species infecting mango cultivars in major growing areas in China, using morphological and molecular techniques together with pathogenicity tests on detached leaves and fruits. Over 200 *Colletotrichum* isolates were obtained across all mango orchards investigated, and 128 of them were selected for sequencing and analyses of actin (ACT), chitin synthase (CHS-1), glyceraldehyde-3-phosphate dehydrogenase (GAPDH), the internal transcribed spacer (ITS) region, β-tubulin (TUB2) genomic regions. Our results showed that the most common fungal isolates associated with mango in southern China involved 13 species: *Colletotrichum asianum*, *C. cliviicola*, *C. cordylinicola*, *C. endophytica*, *C. fructicola*, *C. gigasporum*, *C. gloeosporioides*, *C. karstii*, *C. liaoningense*, *C. musae*, *C. scovillei*, *C. siamense* and *C. tropicale*. The dominant species were *C. asianum* and *C. siamense* each accounting for 30%, and *C. fructicola* for 25%. Only *C. asianum*, *C. fructicola*, *C. scovillei* and *C. siamense* have previously been reported on mango, while the other nine *Colletotrichum* species listed above were first reports associated with mango in China. From this study, five *Colletotrichum* species, namely *C. cordylinicola*, *C. endophytica*, *C. gigasporum*, *C. liaoningense* and *C. musae* were the first report on mango worldwide. Pathogenicity tests revealed that all 13 species caused symptoms on artificially wounded mango fruit and leaves (cv. Tainong). There was no obvious relationship between aggressiveness and the geographic origin of the isolates. These findings will help in mango disease management and future disease resistance breeding.

## Introduction

Mango (*Mangifera indica* L.) is one of the most economically important fruit crops worldwide, and after India, China is the second largest mango producer^1^. The planted area of mango in China was approximately 173,000 ha and the total production was 1,437,700 tons on the mainland in 2014^2^. Major mango producing areas in China include Guangdong, Guangxi, Guizhou, Fujian, Hainan, Yunnan and Sichuan provinces. Mango anthracnose caused by several *Colletotrichum* species, has been considered the most important disease on mango in China^3^. Annually mango anthracnose could be liable for about 30–60% loss of production, and the damage could be 100% if optimum conditions exist for the pathogen^4^.

Traditionally, *Colletotrichum* species are identified based on morphological characters including conidial and appressorial size and shape, presence of acervuli, setae, or sclerotia, sexual structures, and cultural characters such as growth rate and colony color^5^. Distinguishing *Colletotrichum* species within the genus morphologically has been difficult due to their phenotypic similarity, and the fact that variable environmental factors could also affect expression of morphological traits. Frequently, all available morphological characteristics are combined and used for systematic species discrimination^6^.

Studies on *Colletotrichum* have classified the genus into nine major clades plus isolated species or small clusters, with clades probably representing species complexes^7^. The internal transcribed spacer region (ITS) of ribosomal DNA has been frequently used to separate *Colletotrichum* species^8^, but sometimes the ITS sequence information alone was not enough for clarifying species boundaries within the genus^7^. Thus, other genes including ACT, CHS-1, GAPDH and TUB2 have been used to resolve relationships among many fungi, including *Colletotrichum* species^9^. A multiple evidence approach, using combined molecular sequencing information and morphological data is now recommended as an alternative method for accurate species identification in the genus *Colletotrichum*^6,7,9^.

*Colletotrichum* species complexes such as *C. acutatum*, *C. amagnum*, *C. aorchidearum*, *C. boninense*, *C. dracaenophilum* and *C. gloeosporioides* have been separated into many different species using morphological and molecular techniques^9–13^. However, in China the identification of *Colletotrichum* species on mango has relied mainly on conidial morphology and ITS sequences^14^, and the combined data were still limited and insufficient to distinguish closely related taxa in the six species complexes mentioned above. Previous research regarding *Colletotrichum* species on mango were mostly samples from small and fragmented provincial areas, with poor representation of the actual species diversity in the vast mango growing regions in China.

In our previous study, three *Colletotrichum* species causing mango anthracnose in Guangxi province in China (*C*. *asianum*, *C*. *fructicola* and *C*. *siamense*) were reported^15^. However, the growing season and the harvesting time of mango in seven provinces growing mangoes vary in environment, climatic conditions and varieties grown, which may lead to variations in composition and distribution of causal agents of mango anthracnose.

Genetic diversity is often related to evolutionary potential, and an ability to adapt to variable environmental conditions^16^. Based on our current understanding of the gene-for-gene hypothesis, resistance genes lose their effectiveness when faced with complex and evolving pathogen populations. An understanding of the population structure and species distribution of pathogens can provide insights into optimal breeding strategies for durable resistance to anthracnose in mangoes and improved cultural controls.

The objectives of this study were as follows: (1) to identify *Colletotrichum* species associated with mango anthracnose symptoms in six provinces of China using cultural characteristics such as conidial and appressorial morphology as well as multi locus sequencing data for phylogenetic analyses; (2) to test the aggressiveness of isolates from different geographic locations in the major mango growing areas in mainland China; and (3) to examine the variation of isolates within and between regions.

## Results

### Fungal isolations

In 2016 and 2017, all the leaves, fruits and branches of mango with anthracnose symptoms collected from the six mango-growing provinces in China were used for fungal isolation. Based on morphology observation and ITS sequences, a total of 205 *Colletotrichum* isolates were obtained from all mango growing regions investigated, and 128 representative isolates were selected for further study (one isolate from each orchard, and more than one for some big orchards; 34 from Yunnan, 23 from Hainan, 18 from Guizhou, 13 from Sichuan, 16 from Guangdong and 24 from Fujian province). The selected isolates are listed in Supplementary Table S1. Isolated species in other genera include *Alternaria*, *Phomopsis*, *Botryosphaeria*, and *Pestalotia* and were retained for further study.

### Phylogenetic analyses

These isolates from different areas were used for sequencing and analyses of ACT, CHS-1, GAPDH and TUB2 loci. All sequences were first compared with the NR database from NCBI-GenBank using BLASTn, and then submitted to GenBank (see Supplementary Table S1). For phylogenetic analysis, sequences of extype or ex-epitype strains of *Colletotrichum* species (Supplementary Table S2) were selected. According to the ILD test, partitions of the data into ACT, CHS-1, GAPDH, ITS and TUB2 were homogeneous, and the data sets were combined for Maximum likelihood (ML) and Bayesian analyses. For the Bayesian analyses, a JC model was selected for ACT, a GTR + I + G model for GAPDH, ITS and CHS-1, a HKY + G model for TUB2 and incorporated in the analysis.

**Figure 1:**
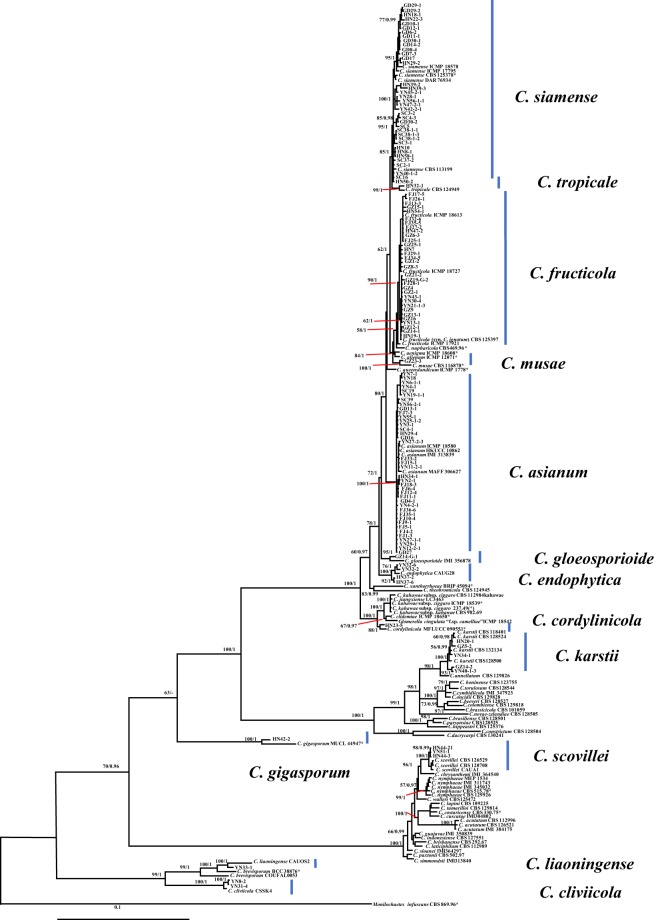
The phylogenetic tree was constructed using sequences of 128 *Colletotrichum* isolates, extype or ex-epitype isolates in the GenBank based on combined ITS, ACT, GAPDH, TUB2, and CHS-1 genomic data. The numbers on the left and right of each node are posterior probabilities estimated using the software MrBayes v. 3.2.6 and bootstrap values from RAxMLGUI v. 1.3.1, respectively.

Concatenated sequences from the five loci (ACT, TUB2, CHS-1, GAPDH and ITS) were generated for each of the 128 *Colletotrichum* isolates, plus ex-type or type strains downloaded from GenBank to generate a phylogenetic tree (Fig. 1). As the topology of the Bayesian analysis of the combined data set was nearly identical to that of the ML consensus tree, only the Bayesian tree is shown with bootstrap (from ML analysis) on the left and posterior probability (from Bayesian analysis) on the right of corresponding nodes (Fig. 1).

The results showed that 38 isolates were clustered with four *C. asianum* strains (ICMP 18605, ICMP 18603, IMI 313839 and ICMP 18580) showing 100% posterior probability (PP) and bootstrap (BS); 38 isolates were aligned with five *C. siamense* strains (with 100% posterior probability and 95% bootstrap); and 32 isolates were placed with two *C. fructicola* strains (ICMP 18727 and ICMP 18613) and one *C. ignotum* strain (CBS125397) supported with 100% posterior probability and 90% bootstrap. Five isolates clustered with strains of *C. karstii* CBS118401, CBS 128524 and CBS 128500, with 100% support; four isolates clustered with the type strain of *C. endophytica* CAUG28 with 100% support; three isolates clustered with *C. scovillei* strains CAUA1, CBS 126529 and CBS 120708 with 100% support, isolates YN8-2 and YN31-4 with type strain *C. cliviicola* CBS 125375 with 100% support; isolate HN42-2 with type strain of *C. gigasporum* CBS 133266, MUCL 44947 with 100% support and isolate HN32-1 with type strain *C. tropicale* CBS 124949 with 100% probability and 99% bootstrap support. Isolate GZ14-G-1 clustered with type strain *C. gloeosporioides* IMI 356878 with 100% probability and 95% bootstrap support, isolate YN33-1 with type strain *C. liaoningense* CAUOS2 with 100% support, HN23-5 with type strain of *C. cordylinicola* ICMP 18579 (100% for PP and 88% for BS) and GZ23-3 with type strain of *C. musae* ICMP 19119 (100%).

### Morphological observations

**Figure 2:**
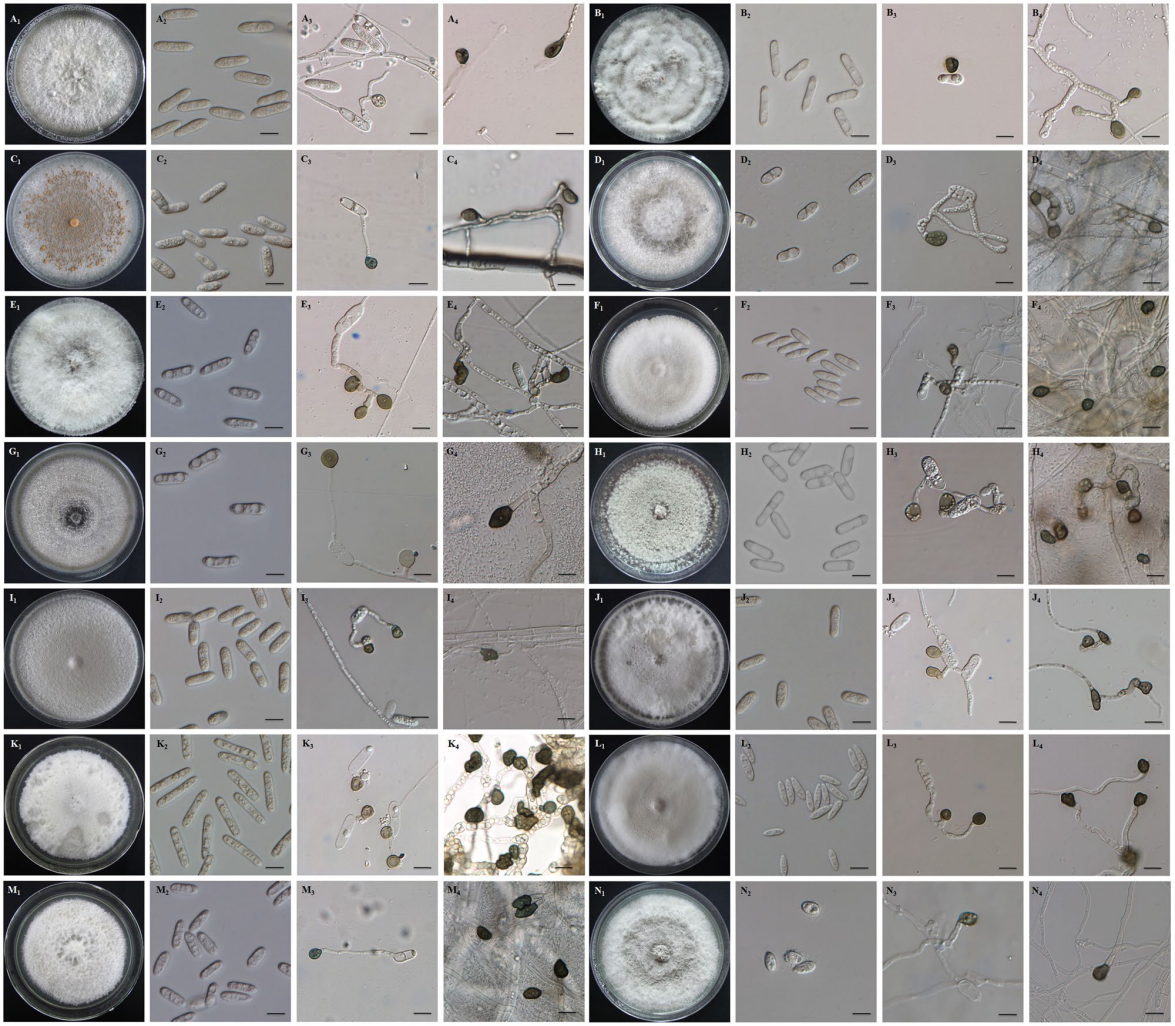
Morphological characteristics of colonies, conidia and appressoria of mango *Colletotrichum* isolates from China. Upper sides of (A1) *C. asianum* FJ18-3, (B1) *C. asianum* FJ31-6, (C1) *C. siamense* HN10, (D1) *C. fruticola* GZ15-1, (E1) *C. karstii* YN34-1, (F1) *C. endophytica* HN37-2, (G1) *C. scovillei* YN51-1, (H1) *C. cliviicola* YN8-2, (I1) *C. gigasporum* HN42-2, (J1) *C. gloeosporioides* GZ14-G-1, (K1) *C. liaoningense* YN33-1, (L1) *C. musae* GZ23-3, (M1) *C. tropicale* YN40-1-2, (N1) *C. cordylinicola* HN23-5 on PDA plates at 14 days after inoculation; Conidia of (A2) *C. asianum* FJ18-3, (B2) *C. asianum* FJ31-6, (C2) *C. siamense* HN10, (D2) *C. fruticola* GZ15-1, (E2) *C. karstii* YN34-1, (F2) *C. endophytica* HN37-2, (G2) *C. scovillei* YN51-1, (H2) *C. cliviicola* YN8-2, (I2) *C. gigasporum* HN42-2, (J2) *C. gloeosporioides* GZ14-G-1, (K2) *C. liaoningense* YN33-1, (L2) *C. musae* GZ23-3, (M2) *C. tropicale* YN40-1-2, (N2) *C. cordylinicola* HN23-5 on PDA plates after 14 days at 25 °C; Conidial appressoria of (A3) *C. asianum* FJ18-3, (B3) *C. asianum* FJ31-6, (C3) *C. siamense* HN10, (D3) *C. fruticola* GZ15-1, (E3) *C. karstii* YN34-1, (F3) *C. endophytica* HN37-2, (G3) *C. scovillei* YN51-1, (H3) *C. cliviicola* YN8-2, (I3) *C. gigasporum* HN42-2, (J3) *C. gloeosporioides* GZ14-G-1, (K3) *C. liaoningense* YN33-1, (L3) *C. musae* GZ23-3, (M3) *C. tropicale* YN40-1-2, (N3) *C. cordylinicola* HN23-5 on glass slides after 3 days at 25 °C; mycelial appressoria of (A4) *C. asianum* FJ18-3, (B4) *C. asianum* FJ31-6, (C4) *C. siamense* HN10, (D4) *C. fruticola* GZ15-1, (E4) *C. karstii* YN34-1, (F4) *C. endophytica* HN37-2, (G4) *C. scovillei* YN51-1, (H4) *C. cliviicola* YN8-2, (I4) *C. gigasporum* HN42-2, (J4) *C. gloeosporioides* GZ14-G-1, (K4) *C. liaoningense* YN33-1, (L4) *C. musae* GZ23-3, (M4) *C. tropicale* YN40-1-2, (N4) *C. cordylinicola* HN23-5 on cover slips after 7 days at 25 °C. Bars: 10 μm.

The colonies of the *Colletotrichum* isolates were white initially, later turning pale gray, with sparse aerial mycelium, hairy, sometimes producing abundant orange (#FF8040) pionnotes (Fig. 2). There was high variation in mycelial growth rate among the 128 *Colletotrichum* isolates (Supplementary Table S3) with averages for *C. asianum* at 0.38–1.59 cm/day, *C. siamense*, 0.52–1.51 cm/day and *C. fructicola*, 0.60–1.19 cm/day.

Conidia were all hyaline, aseptate and long elliptic to cylindrical. The average conidial sizes (Supplementary Table S3) varied among species. For isolates grown on PDA, the length of conidia for each isolate varied from 6.99 μm to 29.22 μm, and the width of conidia varied from 2.99 μm to 9.85 μm (Supplementary Table S3). The mean conidial length and width ranged from 11.5 μm to 25.5 μm and 4.0 μm to 8.2 μm. The conidial morphology of *Colletotrichum* species on mango differed significantly between and within species. For examples, *C. fructicola* (GZ15-1) had profuse sporulation, *C. gigasporum* (HN42-2) produced the largest spores, and the shortest spores were generated by *C. scovillei* (YN51-1).

Conidial appressoria were grey cloud (#B6B6B4) to black (#000000), nearly elliptical or irregular. The length of appressoria for each species varied from 4.9 μm to 14.9 μm and the width of appressoria varied from 3.6 μm and 10.6 μm (Table 1). The mean length and width calculated for each isolate ranged from 6.4 μm to 12.4 μm. Mycelial appressoria were pale brown to dark brown, rod-shaped, occasionally irregular. The length of appressoria for each species varied from 5.6 μm to 20.7 μm and the width of appressoria varied from 3.5 μm to 15.4 μm. The mean value of length and width calculated for each isolate ranged from 7.6 μm to 15.0 μm and 5.5 to 12.2 μm, respectively.

**Table 1:** Appressorial size for 13 *Colletotrichum* species from mango from six provinces in China

Isolates	Size of conidial appressoria (Mean ± SD, μm)^a^	Size of mycelial appressoria (Mean ± SD, μm)
*Colletotrichum assianum* FJ18-3	8.55 ± 0.51 × 7.08 ± 0.24	8.85 ± 0.25 × 6.58 ± 0.13
*C. siamense* HN10	8.41 ± 0.27 × 6.22 ± 0.44	8.26 ± 0.20 × 6.32 ± 0.20
*C. fructicola* GZ15-1	8.39 ± 0.49 × 6.33 ± 0.31	7.78 ± 0.13 × 6.31 ± 0.11
*C. karstii*YN 34-1	11.92 ± 0.51 × 7.35 ± 0.22	7.55 ± 0.40 × 5.66 ± 0.11
*C. endophytica* HN37-2	9.32 ± 0.16 × 7.10 ± 0.18	9.62 ± 0.63 × 5.72 ± 0.26
*C. scovillei* YN51-1	6.38 ± 0.27 × 4.91 ± 0.19	7.88 ± 0.30 × 5.45 ± 0.14
*C. cliviicola* YN8-2	8.94 ± 0.29 × 7.44 ± 0.28	11.15 ± 0.94 × 7.89 ± 0.40
*C. gigasporum* HN42-2	12.41 ± 0.41 × 9.36 ± 0.34	14.99 ± 0.39 × 12.21 ± 0.34
*C. gloeosporioides* GZ14-G-1	8.81 ± 0.41 × 5.26 ± 0.19	10.51 ± 0.78 × 5.94 ± 0.24
*C. liaoningense* YN33-1	7.03 ± 0.15 × 6.00 ± 0.12	10.47 ± 0.34 × 7.61 ± 0.19
*C. musae* GZ23-3	6.71 ± 0.21 × 6.09 ± 0.21	8.68 ± 0.30 × 6.63 ± 0.19
*C. tropicale* YN40-1-2	7.94 ± 0.21 × 6.98 ± 0.22	10.41 ± 0.44 × 6.62 ± 0.16
*C. cordylinicola* HN23-5	8.22 ± 0.21 × 5.15 ± 0.18	10.51 ± 0.47 × 6.44 ± 0.27

^a^.The length and width of 30 conidial appressoria and mycelial appressoria per isolate on glass and plastic slides were measured after 3 days and 7 days at 25 °C, respectively.

The various *Colletotrichum* species reported here showed differences in both conidial and appressorial sizes and growth rates (Table 1 and Supplementary Table S3). Based on morphology observation and multi loci sequence analysis, 128 mango *Colletotrichum* isolates obtained in China can be classified into 13 species as follow: 38 isolates (29.7%) *C. asianum*, 38 isolates (29.7%) *C. siamense*, 32 isolates (25.0%) *C. fructicola*, five isolates *C. karstii* (3.9%), four isolates *C. endophytica* (3.1%), three isolates *C. scovillei* (2.3%), two isolates *C. cliviicola* (1.6%) and one isolate for each of the following species: *C. cordylinicola, C. gigasporum*, *C. gloeosporioides*, *C. liaoningense*, *C. musae* and *C. tropicale*.

This is the first report for *C. cliviicola*, *C. cordylinicola*, *C. endophytica*, *C. gigasporum*, *C. gloeosporioides*, *C. karstii*, *C. liaoningense*, *C. musae* and *C. tropicale* associated with mango anthracnose in China. This is also the first report for *C. cordylinicola*, *C. endophytica*, *C. gigasporum*, *C. liaoningense* and *C. musae* associated with mango anthracnose worldwide.

### Provincial geographic distribution of *Colletotrichum* species on mango in China

**Figure 3:**
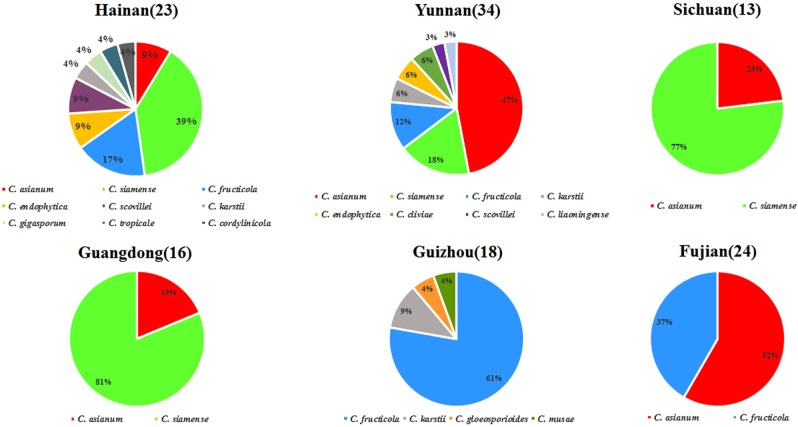
The percentage (%) for dominant *Colletotrichum* species on mango isolated from Hainan, Yunnan, Sichuan, Guizhou, Guangdong and Fujian provinces in China with number of isolates from each province.

The diversity of species and geographical distribution for various *Colletotrichum* species associated with mango anthracnose across different provinces in China are shown in Fig. 3. Hainan and Yunnan had the most abundant and diverse *Colletotrichum* species on mango. Nine *Colletotrichum* species (*C. asianum*, *C. cordylinicola*, *C. endophytica*, *C. fructicola*, *C. gigasporum*, *C. karstii*, *C. scovillei*, *C. siamense*, and *C. tropicale*) were recovered from mango with anthracnose symptoms in Hainan province while eight species (*C. asianum*, *C. cliviicola*, *C. endophytica*, *C. fructicola*, *C. karstii*, *C. liaoningense*, *C. scovillei* and *C. siamense*) were obtained from mango in Yunnan. Four species (*C. fructicola*, *C. gloeosporioides*, *C. karstii* and *C. musae*) were associated with mango anthracnose in Guizhou province. Only two *Colletotrichum* species (*C. asianum* and *C. siamense*) were isolated in Sichuan and Guangdong provinces. *C. asianum* and *C. fructicola* were found in Fujian province. As a whole, *C. asianum*, *C. fructicola*, *C. siamense* were the dominant species widely spread across the major mango growing areas in southern China, accounting for 84.4% of all isolates. *C. endophytica*, *C. gigasporum, C. gloeosporioides* and *C. liaoningense* were only found on wild mango trees (Supplementary Table S1).

### Pathogenicity and aggressiveness in tissues

All 128 tested isolates were pathogenic to wounded mango leaves and fruits. The aggressiveness of the tested isolates on mango fruits differed significantly, with lesion diameters ranging from 0.54 cm to 3.64 cm (Supplementary Table S3). Based on diameter sizes of the disease spots, aggressiveness was classified as strongly virulent ($\underline{\underline{ > }}$2 cm), moderately virulent ($\underline{\underline{ > }}$1.0 to <2 cm) or weakly virulent (<1.0 cm). Among the 128 isolates investigated, 66 (52%) isolates were moderately virulent, 9 (7%) isolates were strongly virulent, 53 (41%) isolates were weakly virulent. The least virulent strain was *C. siamense* SC16, while the most virulent strain was *C. siamense* GD7-1 (Supplementary Table S3).

The aggressiveness of the tested isolates on mango leaves was also significantly different, and the diameters of disease lesions varied from 0.60 cm to 4.34 cm (Supplementary Table S3). The aggressiveness of the tested strains in mango differed considerably. According to the diameter size of the disease lesions, the aggressiveness scale can be classified as strongly virulent ($\underline{\underline{ > }}$2 cm), moderately virulent ($\underline{\underline{ > }}$1.0 to <2 cm) and weakly virulent (<1.0 cm). Using this scale, 84 isolates (65%) were moderately virulent, 38 isolates (30%) were strongly virulent, 6 isolates (5%) were weakly virulent. The least virulent strain was *C. siamense* YN56-1-1, while the most virulent strain was *C. siamense* SC2-1 (Supplementary Table S3).

## Discussion

In the past, common identification systems in *Colletotrichum* included traditional morphological taxonomy, molecular classification based on ITS sequences, presence of secondary metabolites and some other traits. These previously used identification systems alone or in combination were unable to fully resolve the species identity of individual *Colletotrichum* isolates^6^. Additional characteristics such as other aspects of morphology, and multi-gene phylogenetic analyses are needed to identify *Colletotrichum* species^6^.

Based on morphology and a concatenated five-gene phylogenetic analysis, 128 *Colletotrichum* isolates on mango from southern China were separated into 13 species: *C. asianum*, *C. cliviicola*, *C. cordylinicola*, *C. endophytica*, *C. fructicola*, *C. gigasporum*, *C. gloeosporioides*, *C. karstii*, *C. liaoningense*, *C. musae*, *C. scovillei*, *C. siamense*, and *C. tropicale*. Four species complexes (*C. acutatum*, *C. boninense*, *C. gigasporum* and *C. gloeosporioides*) were found to be associated with mango. The morphological descriptions of the isolates were in agreement with previous descriptions of *Colletotrichum* species, although cultural characteristics may be diverse due to varied cultural conditions, and conidial morphology alone could not distinguish all species within species complexes^9^.

Currently, thirteen C*olletotrichum* species have been reported worldwide on mango: *C*. *asianum*^17^, *C*. *cliviicola*^18^, *C*. *dianesei*^18^, *C*. *endomangiferae*^18^, *C*. *fructicola*^17^, *C. gloeosporioides*^19^, *C. grossum*^20^, *C*. *kahawae*^21^, *C*. *karstii*^18^, *C. scovillei*^22^, *C*. *siamense*^23^, *C. theobromicola* (syn. *C. fragariae*)^19^ and *C*. *tropicale*^18^. Mo *et al*.^15^, Liu *et al*.^24^ and Qin *et al*. ^22,25^ identified four *Colletotrichum* species (*C. asianum*, *C. fructicola*, *C. scovillei*, *C. siamense*) prevalent on mango in China. In this study, however, aside from *C. asianum*, *C. fructicola*, *C. scovillei* and *C. siamense*, there were 9 *Colletotrichum* species (*C. cliviicola*, *C. cordylinicola*, *C. endophytica*, *C. gigasporum*, *C. gloeosporioides*, *C. karstii*, *C. liaoningense*, *C. musae*, *C. tropicale*) which are the first reports on mango in China. This research also provides first reports for *C. gigasporum*, *C. cordylinicola*, *C. musae*, *C. liaoningense* and *C. endophytica* associated with mango world-wide.

The present study revealed high *Colletotrichum* species diversity associated with mango from the six major mango-grown areas in China. The *Colletotrichum* isolates examined here also showed high diversity, which may correlate with the diversity of environmental conditions such as temperature and rainfall^26^ and the sample collection time and isolate source. *Colletotrichum asianum*, *C. fructicola* and *C. siamense* were found to be the major species associated with mango anthracnose in China. This result agrees with what was reported by Vieira *et al*.^18^ (2014) who found that *C. asianum* was the most common endophytic species from mango trees. *Colletotrichum asianum* and *C. siamense*, were report as pathogens of a wide diversity of hosts such as *Coffea arabica*^9^, *Carica papaya*^9^ and *M. indica*^15,19,27^. Our study showed that a single host species may be attacked by several *Colletotrichum* species, and this was previously reported by Phoulivong *et al*.^28^. *Colletotrichum fructicola* was initially reported on coffee berries, and it also has a wide host range (*Arachis* sp., *Citrus bergamia* and *M. indica*) as well as wide geographical distribution^29^. Similarly, *C. tropicale* has been reported on *Annona muricata* (Annonaceae), *Viola surinamensis* (Myristicaceae) and *M. indica* in Brazil^29^.

*Colletotrichum musae* is a major causal agent of banana anthracnose and associated with fruit disease spots for *Musa* sp. in many countries^29^. *Colletotrichum cliviicola* was found to be associated with anthracnose of *Clivia miniata, Cymbidium hookerianum* in China^12,30^ and of *Phaseolus* sp. and *Saccharum* sp. in India^12^. It was also reported as an endophyte on *M. indica* in Brazil^18^.

*Colletotrichum gloeosporioides* was reported as a main pathogen from *Citrus* sp., *Mangifera* sp., and *Vitis vinifera* among many other host species, since it is considered a species complex^9^. It has a particularly wide host range based on publications because many *Colletotrichum* isolates were previously labelled *C. gloeosporioides*^29^. In the present study, *C. asianum*, *C. cordylinicola*, *C. fructicola*, *C. gloeosporioides*, *C. kahawae*, *C. musae*, *C. siamense* and *C. tropicale*, can all be placed into the *C. gloeosporioides* species complex^9^, and these results are consistent with previous reports that the *C. gloeosporioides* species complex is a preponderant *Colletotrichum* species associated with mango anthracnose^23,31^.

*Colletotrichm scovillei* was reported as a pathogen on mango^22^ and *Capsicum* sp. (chilli “Django”)^32^, and has been placed into the *C. acutatum* species complex^10^. *Colleotrichum karstii* was first reported from orchids and was also known from some other hosts such as *Carica papaya*^11^, *Citrus* sp.^11^, and *M. indica*^11,17^. *Colletotrichum karstii* has been found on mango and avocado fruit^17,33^, and it has been placed in the *C. boninense* species complex^11^.

*Colletotrichum gigasporum* belongs to the *C. gigasporum* species complex^13^. The species has large conidia and its wide range of hosts include *Acacia auriculiformis* and *Coffea* sp. (in Vietnam), and *Diospyros kaki* and *Musa* sp. (in Japan). *Colletotrichum endophytica* was found on *Pennisetum purpureum* in Thailand and tea plant in China^34^. *Colletotrichum liaoningense* was first reported as a pathogen of *Capsicum annuum* var. *conoides* in China^35^.

Pathogenicity test using thirteen species of *Colletotrichum* isolates showed that all species were pathogenic to mango leaves and fruits in the wound-inoculation experiment. The pathogenicity tests indicated that aggressiveness of *Colletotrichum* species on fruit was not completely consistent to that on leaves, but the aggressiveness of different isolates was found to be significantly different. The difference in aggressiveness of *Colletotrichum* within species was independent of a particular geographical origin for sample collection and varied, perhaps resulting from adaptations to the geographic and environmental origins of the isolates, although no specific correlations were found between aggressiveness and province of origin. However, there were more isolates from Yunnan province with weak aggressiveness compared to those from Hainan province. Symptoms may vary considerably with factors such as cultivar, fruit physiological state, inoculum factors such as concentration, and environmental conditions such as humidity and temperature^33,36,37^. Thus, the pathogenicity test results from this study may not reflect the full aggressiveness potential of the isolates examined. Additional research should be conducted to determine the aggressiveness for *Colletotrichum* species with conidial inoculation in planta rather than wounded detached leaves and fruits with mycelial plugs.

This study has enhanced our understanding on the diversity of *Colletotrichum* species associate with mango anthracnose in China. Further study is required to determine differences in biological and infection characteristics among various mango *Colletotrichum* species, as well as the molecular mechanisms responsible for such differences and their effects on mango anthracnose disease in the field. There have been few studies on the etiology and epidemiology of *Colletotrichum* species on mango. Therefore, future work on pathogenic mechanisms could be helpful for prevention and control of mango anthracnose disease.

## Materials and Methods

### Collection of sample and isolation of fungi

During 2016 and 2017, mango leaves, fruits and branches with symptoms or signs of anthracnose were gathered from six provinces (Fujian, Guangdong, Guizhou, Hainan, Sichuan and Yunnan) in China, covering an area 1950 km by 800 km. Mango orchards were sampled with minimum 2 km distance between samples. Lesion margins on mango leaves, branches and fruits were cut into 3 mm × 3 mm pieces, surface sterilized with ethanol (75%) for 15 s, sodium hypochlorite (1% vol/vol) for 2 min, followed by three rinses in autoclaved distilled water for 30 s. Isolated fungus were cultured on potato dextrose agar (PDA) and kept at 25 °C. Over two weeks, the cultures were examined every day. Isolates were placed on PDA slants for longer term storage at 4 °C in the dark.

### Morphological characterization

Before morphological examination, isolates were cultured under continuous fluorescent light on PDA for week at 25 °C. Mycelial plugs (5 mm diam) were cut from colony margins and placed 9-cm-diam Petri dishes with four plates per isolate. The colony characteristics (color of upper and lower surfaces) plus growth rate (diameters in two perpendicular directions) were recorded after 3, 5, 7 and 10 days. Hyphal growth rate (cm/day) was calculated based on colony diameter. Conidial production, shape and the size of conidia were examined up to three weeks on PDA incubated at 25 °C. Conidia (120 per isolate) from three-week-old PDA cultures were measured for length and width. Appressoria were produced from conidia placed on glass slides inside moist chambers. Appressoria were produced from hyphae using a slide culture technique described by Cai *et al*.^6^. PDA plugs (1 cm × 1 cm) were placed in clean Petri dishes, and each agar plug was treated with conidia, and a cover slip was placed over each plug. After 7 days, the shape and size of the appressoria were recorded. For each isolate, at least 30 appressoria were examined, and photographs were taken under a Nikon Eclipse Ni-E microscope.

### DNA extraction, polymerase chain reaction (PCR), and sequencing

Isolates were incubated at 25 °C on PDA in Petri plates overlaid with cellophane. After 7 days of growth, mycelia were harvested by scraping the cellophane with autoclaved spatulas, and DNA was extracted using a CTAB method^38^. DNA was amplified with primers for partial actin (ACT), partial chitin synthase (CHS-1), partial glyceraldehyde-3-phosphate dehydrogenase (GAPDH), rDNA-ITS (ITS), and partial β-tubulin (TUB2) genomic regions (Supplementary Table S4). PCR amplification of ACT, CHS-1, GAPDH, TUB2 and ITS was amplified with the primer-pairs shown in Supplementary Table S4. PCR amplifications were done in 50-μl volumes containing 4 mM MgCl_2_, 1 × PCR buffer, 0.5 units Taq DNA polymerase (Takara), 0.2 mM concentrations of each dNTP, 0.5 μM concentrations of each primer and 1 μl of template DNA (20 ng/μl). The PCR programs for ACT, CHS-1, GAPDH, ITS and TUB2 were set following Mo *et al*.^15^ The PCR products were sequenced by Shanghai Sangon Company in China.

### Phylogenetic analyses

Consensus sequences were obtained from forward and reverse primer sequencing of the same genomic region for each sequenced isolate using DNAMAN version 7.0. The consensus sequences were compared by BLASTn^39^ against the NCBI NR database. Among the top matches, select sequences of extype or ex-epitype isolates of *Colletotrichum* species were selected for the phylogenetic analyses (Supplementary Table S2). Alignments for each gene were carried out using MUSCLE v. 3.8.3^40^. We tested whether the five gene data sets were combinable using the Partition Homogeneity Test as implemented in PAUP* 4.0b4 and described by Farris *et al*.^41^ as the incongruence length difference (ILD) test^41^. Sequences from the five genes were then manually concatenated and re-aligned. Phylogenetic analysis was conducted using MrBayes version 3.2.6^42^. Models of nucleotide substitution for each gene determined by jModeltest v. 2.1.7 were included for each gene partition^43^. Following Drummond and Rambaut^44^ four runs with four chains for 5,000,000 generations were set up while the first 25% were discarded. The analyses were sampled every 1000 generations until the average standard deviation of split frequencies fell below 0.01. Maximum likelihood analyses were conducted with the RAxML GUI v. 1.3.1^45^ using a GTRGAMMA substitution model, and bootstrap support was calculated based on 1,000 iterations.

### Pathogenicity and aggressiveness on mango tissues

Pathogenicity tests were conducted following Mo *et al*.^15^. Select isolates were used for pathogenicity and aggressiveness tests on detached leaves and fruits of mango (cv. Tainong) under controlled conditions. Each isolate was incubated on PDA for 7–10 days at 25 °C. Freshly harvested mango fruits and young leaves without visible disease from Tiandong (Guangxi province) were used for the tests. The tissues were surface sterilized in 75% ethanol for 30 s and 1% NaClO for 1 min, and then with three rinses in autoclaved distilled water.

After air drying, detached young healthy leaves (12–15 cm) were placed into plastic containers (9 cm × 17 cm × 25 cm) lined with paper towelling, and six stab wounds were made forming a 5-mm-diam circle. Mycelial plugs (6 mm diameter) from margins of PDA cultures were placed on each wound. The completely randomized trial with three replicates per isolate. For controls, sterile agar plugs were used. The containers were sealed and incubated in the dark at 25 °C. Aggressiveness was assessed one week after inoculation by measuring lesion diameter.

After washing and air drying, newly ripened mango fruits (∼100 g) were wounded by stabbing to a depth of 3 mm to form a 5-mm-diam circle. Mycelial plugs (6 mm diameter) from PDA cultures were placed on the wounds. Sterile agar plugs were used as control treatments. Inoculated fruits were placed in plastic containers on top of moist paper towelling and sealed. The containers were placed in a growth chamber and incubated at 25 °C in the dark. Symptom development of the fruits was checked daily up to 10 days. Isolates were considered pathogenic when lesions expanded beyond the 5 mm circle of the initial wounding. Aggressiveness was evaluated at 10 days after inoculation (dpi) by measuring lesion diameter.

### Statistical analyses

Statistical analyses were done using Data Processing System software (DPS 7.0) for Windows (Zhejiang University, Hangzhou, China)^46^, and data were subjected to analysis of variance (ANOVA) followed by Fisher’s Least Significant Difference” (LSD) tests for means separation.

## Supplementary information

Supplementary information

## Data Availability

All data can be available to Editorial Board Members, referees and readers.
