# Supplementary material for: Colletotrichum species associated with mango in southern China
Source: Sci Rep. 2019 Dec 11;9:18891. doi: 10.1038/s41598-019-54809-4 (PMC6906457; doi:10.1038/s41598-019-54809-4)
Supplement: Supplementary file 1 — Supplementary information [file 41598_2019_54809_MOESM1_ESM.docx]

**Supplementary information**

***Colletotrichum* species associated with mango in southern China**

**Qili Li^1^**, **Junyan Bu^2^**, **Juan Shu^2^**, **Zhihe Yu^2^**, **Lihua Tang^1^**, **Suiping Huang^1^**, **Tangxun Guo^1^**, **Jianyou Mo^1^**, **Shuming Luo^3^**, **Ghulam Sarwar Solangi^4^**, and **Tom Hsiang^5^**

1 Institute of Plant Protection, Guangxi Academy of Agricultural Sciences and Guangxi Key Laboratory of Biology for Crop Diseases and Insect Pests, Nanning, Guangxi, 530007, China;

2 College of Life Sciences, Yangtze University, Jingzhou, Hubei, 434025, China;

3 Plant Breeding Institute, The University of Sydney, Cobbitty, NSW 2570, Australia;

4 Department of Entomology, Sindh Agriculture University Sub-campus, Umerkot 69100, Pakistan;

5 Environmental Sciences, University of Guelph, Guelph, Ontario, N1G 2W1, Canada.

Dr. Zhihe Yu, email: zhiheyu@hotmail.com and Prof. Jianyou Mo, email: mojianyou@gxaas.net

**Supplementary Table S1** List of *Colletotrichum* species, isolate number, GenBank accession number and their geographic origin collected in this study

| Species | Isolate | Geographic area | variety | Isolation source | GenBank accession numbers 1 | | | | |
| --- | --- | --- | --- | --- | --- | --- | --- | --- | --- |
|  |  |  |  |  | ACT | CHS-1 | GAPDH | ITS | TUB2 |
| *C. asianum* | GD4-1 | Dongan, Changqi, Huazhou, Guangdong | unknown | leaf | MH622349 | MH622485 | MH681290 | MH636411 | MH622621 |
| *C. asianum* | GD16 | Xuwu, Nanting, Suixi, Zhanjiang, Guangdong | Tainong | leaf | MH622358 | MH622494 | MH681299 | MH636420 | MH622630 |
| *C. asianum* | GD27 | Hongmaofeng,Wushi, Leizhou, Zhanjiang, Guangdong | Tainong | leaf | MH622360 | MH622496 | MH681301 | MH636422 | MH622632 |
| *C. asianum* | HN29-4 | Haipo, Jiusuo, Ledong, Hainan | Tainong | leaf | MH622393 | MH622529 | MH681330 | MH636451 | MH622661 |
| *C. asianum* | HN34-1 | Shilu, Changjiang, Hainan | wild-unkown | leaf | MH622410 | MH622546 | MH681347 | MH636468 | MH622678 |
| *C. asianum* | SC4-1 | Shitou,Yakou, Miyi, Sichuan | unknown | leaf | MH622414 | MH622550 | MH681351 | MH636472 | MH622682 |
| *C. asianum* | SC19 | Ashuda, Panzhihua, Sichuan | Kate | leaf | MH622418 | MH622554 | MH681355 | MH636476 | MH622686 |
| *C. asianum* | SC39 | Zongfa, Renhequ, Panzhihua, Sichuan | Hongxiangya | leaf | MH622423 | MH622559 | MH681360 | MH636481 | MH622691 |
| *C. asianum* | YN2-1 | Wuxin, Xinzhuang, Rongjiang, Huaping, Yunnan | Kate | leaf | MH622424 | MH622560 | MH681361 | MH636482 | MH622692 |
| *C. asianum* | YN3-1 | Yeshishanzhuang, Xinzhuang, Rongjiang, Huaping, Yunnan | Kate | leaf | MH622425 | MH622561 | MH681362 | MH636483 | MH622693 |
| *C. asianum* | YN4-1 | Wuxin, Xinzhuamg, Rongjiang, Huaping, Yunnan | Kate | leaf | MH622426 | MH622562 | MH681363 | MH636484 | MH622694 |
| *C. asianum* | YN6-1-1 | Hejiawan, Xinzhuang, Rrongjiang, Huaping, Yunnan | Kate | leaf | MH622427 | MH622563 | MH681364 | MH636485 | MH622695 |
| *C. asianum* | YN7-1 | Hejiawan, Xinzhuang, Rrongjiang, Huaping, Yunnan | Kate | leaf | MH622428 | MH622564 | MH681365 | MH636486 | MH622696 |
| *C. asianum* | YN11-2-1 | Heai, Rongjiang, Huaping, Yunnan | Kate | leaf | MH622430 | MH622566 | MH681367 | MH636488 | MH622698 |
| *C. asianum* | YN12-2-1 | Heai, Rongjiang, huaping, Yunnan | Kate | leaf | MH622431 | MH622567 | MH681368 | MH636489 | MH622699 |
| *C. asianum* | YN4-2-1 | Wuxin, Xinzhuamg, Rongjiang, Huaping, Yunnan | Kate | leaf | MH622433 | MH622569 | MH681370 | MH636491 | MH622701 |
| *C. asianum* | YN18 | Pingzi, Rongjiang, Huaping, Yunnan | Yingzui | leaf | MH622434 | MH622570 | MH681371 | MH636492 | MH622702 |
| *C. asianum* | YN19-1-1 | Zheli, Rongjiang, Huaping, Yunnan | Yingzui | leaf | MH622435 | MH622571 | MH681372 | MH636493 | MH622703 |
| *C. asianum* | YN27-2-3 | Hongxinfenchang, Namaluosizhai, Ganzhuang, Yuanjiang, Yunnan | Tainong | leaf | MH622438 | MH622574 | MH681375 | MH636496 | MH622706 |
| *C. asianum* | YN56-2-1 | Minzufengqingyuan, Xishuangbanna, Yunnan | Hongxiangya | leaf | MH622440 | MH622576 | MH681377 | MH636498 | MH622708 |
| *C. asianum* | YN25-1-2 | Hongxinfenchang, Namaluosizhai, Ganzhuang, Yuanjiang, Yunnan | Tainong | leaf | MH622442 | MH622578 | MH681379 | MH636500 | MH622710 |
| *C. asianum* | YN55-1 | Yuanshisenlingongyuan, Xishuangbanna, Yunnan | unknown | leaf | MH622445 | MH622581 | MH681382 | MH636503 | MH622713 |
| *C. asianum* | YN29-1 | Hongxinfenchang, Namaluosizhai, Ganzhuang, Yuanjiang, Yunnan | Tainong | leaf | MH622447 | MH622583 | MH681384 | MH636505 | MH622715 |
| *C. asianum* | YN27-1-1 | Hongxinfenchang, Namaluosizhai, Ganzhuang, Yuanjiang, Yunnan | Tainong | leaf | MH622452 | MH622588 | MH681389 | MH636510 | MH622720 |
| *C. asianum* | FJ1-3 | Senlingongyuan, Tianzhushan, Xiamen, Fujian | unknown | leaf | MH622458 | MH622594 | MH681395 | MH636516 | MH622726 |
| *C. asianum* | FJ4-2 | Hongjiangzhongxue, Zhangpu, Fujian | unknown | leaf | MH622459 | MH622595 | MH681396 | MH636517 | MH622727 |
| *C. asianum* | FJ5-1 | Rongjiang, Zhangpu, Fujian | Tainong | leaf | MH622460 | MH622596 | MH681397 | MH636518 | MH622728 |
| *C. asianum* | FJ6-4 | Daling, Yunling, Yunxiao, Fujian | Tainong | leaf | MH622461 | MH622597 | MH681398 | MH636519 | MH622729 |
| *C. asianum* | FJ7-3 | Nanshan, Puomei, Yunxiao, Fujian | unknown | leaf | MH622462 | MH622598 | MH681399 | MH636520 | MH622730 |
| *C. asianum* | FJ9-1 | Daling, Yunling, Yunxiao, Fujian | unknown | leaf | MH622463 | MH622599 | MH681400 | MH636521 | MH622731 |
| *C. asianum* | FJ10-4 | Nanshan, Puomei, Yunxiao, Fujian | wild-unkown | leaf | MH622464 | MH622600 | MH681401 | MH636522 | MH622732 |
| *C. asianum* | FJ11-1 | Miyuanshengtainongchang, Qianting, Zhangpu, Fujian | Yuwen | leaf | MH622465 | MH622601 | MH681402 | MH636523 | MH622733 |
| *C. asianum* | FJ12-4 | Miyuanshengtainongchang, Qiantign, Zhangpu, Fujian | Yuwen | leaf | MH622466 | MH622602 | MH681403 | MH636524 | MH622734 |
| *C. asianum* | FJ15-1 | Miyuanshengtainongchang, Qiantign, Zhangpu, Fujian | Jinhuang | leaf | MH622468 | MH622604 | MH681405 | MH636526 | MH622736 |
| *C. asianum* | FJ18-3 | Miyuanshengtainongchang, Qiantign, Zhangpu, Fujian | Jinhuang | leaf | MH622470 | MH622606 | MH681407 | MH636528 | MH622738 |
| *C. asianum* | FJ33-2 | Huangqin, Guoken, Zhangzhou, Fujian, | unknown | leaf | MH622480 | MH622616 | MH681414 | MH636535 | MH622745 |
| *C. asianum* | FJ35-1 | Wuzhai, Pinghe, Fujian | unknown | leaf | MH622482 | MH622618 | MH681416 | MH636537 | MH622747 |
| *C. asianum* | FJ36-6 | Xintian, Banzhai, Pinghe, Fujian | unknown | leaf | MH622484 | MH622620 | MH681418 | MH636539 | MH622749 |
| *C. siamense* | GD6-2 | Ganbiao, Xinan, Huazhou, Guangdong | unknown | leaf | MH622350 | MH622486 | MH681291 | MH636412 | MH622622 |
| *C. siamense* | GD7-1 | South Subtropical Crop Research Institute, China Academy of Tropical Agricultural, Guangdong | Tainong | leaf | MH622351 | MH622487 | MH681292 | MH636413 | MH622623 |
| *C. siamense* | GD8-1 | South Subtropical Crop Research Institute, China Academy of Tropical Agricultural, Guangdong | Tainong | leaf | MH622352 | MH622488 | MH681293 | MH636414 | MH622624 |
| *C. siamense* | GD10-1 | South Subtropical Crop Research Institute, China Academy of Tropical Agricultural, Guangdong | Tainong | leaf | MH622353 | MH622489 | MH681294 | MH636415 | MH622625 |
| *C. siamense* | GD11-1 | South Subtropical Crop Research Institute, China Academy of Tropical Agricultural, Guangdong | Tainong | leaf | MH622354 | MH622490 | MH681295 | MH636416 | MH622626 |
| *C. siamense* | GD12-1 | South Subtropical Crop Research Institute, China Academy of Tropical Agricultural, Guangdong | Tainong | leaf | MH622355 | MH622491 | MH681296 | MH636417 | MH622627 |
| *C. siamense* | GD13-1 | Shuanggang, Chikan, Zhanjiang, Guangdong | unknown | leaf | MH622356 | MH622492 | MH681297 | MH636418 | MH622628 |
| *C. siamense* | GD14-2 | Chikan, Zhanjiang, Guangdong | unknown | leaf | MH622357 | MH622493 | MH681298 | MH636419 | MH622629 |
| *C. siamense* | GD17 | Nanting, Suixi, Zhanjiang, Guangdong | unknown | leaf | MH622359 | MH622495 | MH681300 | MH636421 | MH622631 |
| *C. siamense* | GD29-1 | Hongmaofeng, Wushi, Leizhou, Zhanjiang, Guangdong | Danmang | leaf | MH622361 | MH622497 | MH681302 | MH636423 | MH622633 |
| *C. siamense* | GD29-2 | Hongmaofeng, Wushi, Leizhou, Zhanjiang, Guangdong | Danmang | leaf | MH622362 | MH622498 | MH681303 | MH636424 | MH622634 |
| *C. siamense* | GD30-2 | Hongmaofeng, Wushi, Leizhou, Zhanjiang, Guangdong | Tainong | leaf | MH622363 | MH622499 | MH681304 | MH636425 | MH622635 |
| *C. siamense* | GD30-1 | Hongmaofeng, Wushi, Leizhou, Zhanjiang, Guangdong | Tainong | leaf | MH622364 | MH622500 | MH681305 | MH636426 | MH622636 |
| *C. siamense* | HN10 | Nanxingnongchang, Yalongwan, Sanya, Hainan | wild-unkown | leaf | MH622389 | MH622525 | MH681326 | MH636447 | MH622657 |
| *C. siamense* | HN18-1 | Nanbinnongchang, Yacheng, Sanya, Hainan | Tainong | leaf | MH622390 | MH622526 | MH681327 | MH636448 | MH622658 |
| *C. siamense* | HN29-2 | Haipo, Jiusuo, Ledong, Hainan | Tainong | leaf | MH622392 | MH622528 | MH681329 | MH636450 | MH622660 |
| *C. siamense* | HN39-2 | No. 3 Century old Mango tree, Chahe, Changjiang, Hainan | wild-unkown | leaf | MH622396 | MH622532 | MH681333 | MH636454 | MH622664 |
| *C. siamense* | HN39-3 | No. 3 Century old Mango tree, Chahe, Changjiang, Hainan | wild-unkown | leaf | MH622397 | MH622533 | MH681334 | MH636455 | MH622665 |
| *C. siamense* | HN50-2 | Century old Mango tree, Gancheng, Dongfang, Hainan | wild-unkown | leaf | MH622401 | MH622537 | MH681338 | MH636459 | MH622669 |
| *C. siamense* | HN58-1 | Hongxie, Yingchuan, Lingshui, Sanya, Hainan | unkown | leaf | MH622403 | MH622539 | MH681340 | MH636461 | MH622671 |
| *C. siamense* | HN8-1 | Nanxingnongchang, Yalongwan, Sanya, Hainan | Jinhuang | leaf | MH622404 | MH622540 | MH681341 | MH636462 | MH622672 |
| *C. siamense* | HN22-3 | Ducun,Yacheng, Sanya, Hainan | Jinhuang | leaf | MH622407 | MH622543 | MH681344 | MH636465 | MH622675 |
| *C. siamense* | SC2-1 | Yakou, Yakou, Miyi, Sichuan | unknown | leaf | MH622411 | MH622547 | MH681348 | MH636469 | MH622679 |
| *C. siamense* | SC3-1 | Shitou, Yakou, Miyi, Sichuan | unknown | leaf | MH622412 | MH622548 | MH681349 | MH636470 | MH622680 |
| *C. siamense* | SC3-2 | Shitou,Yakou,Miyi, Sichuan | unknown | leaf | MH622413 | MH622549 | MH681350 | MH636471 | MH622681 |
| *C. siamense* | SC4-3 | Shitou,Yakou,Miyi, Sichuan | unknown | leaf | MH622415 | MH622551 | MH681352 | MH636473 | MH622683 |
| *C. siamense* | SC5 | Zaozilin,Yakou,Miyi, Sichuan | unknown | leaf | MH622416 | MH622552 | MH681353 | MH636474 | MH622684 |
| *C. siamense* | SC16 | Ashuda, Panzhihua, Sichuan | Jilu | leaf | MH622417 | MH622553 | MH681354 | MH636475 | MH622685 |
| *C. siamense* | SC37-2 | Zongfa, Renhequ, Panzhihua, Sichuan | Hongxiangya | leaf | MH622419 | MH622555 | MH681356 | MH636477 | MH622687 |
| *C. siamense* | SC38-1-1 | Zongfa, Renhequ, Panzhihua, Sichuan | Hongxiangya | leaf | MH622420 | MH622556 | MH681357 | MH636478 | MH622688 |
| *C. siamense* | SC38-1-2 | Zongfa, Renhequ, Panzhihua, Sichuan | Hongxiangya | leaf | MH622421 | MH622557 | MH681358 | MH636479 | MH622689 |
| *C. siamense* | SC38-1-3 | Zongfa, Renhequ, Panzhihua, Sichuan | Hongxiangya | leaf | MH622422 | MH622558 | MH681359 | MH636480 | MH622690 |
| *C. siamense* | YN45-2-1 | Chengdongdongwuyuan, Xishuangbana, Yunnan | unknown | leaf | MH622436 | MH622572 | MH681373 | MH636494 | MH622704 |
| *C. siamense* | YN28-1 | Hongxinfenchang, Namaluosizhai, Ganzhuang, Yuanjiang | Tainong | leaf | MH622437 | MH622573 | MH681374 | MH636495 | MH622705 |
| *C. siamense* | YN56-1-1 | Minzufengqingyuan, Xishuangbanna, Yunnan | Xiangya | leaf | MH622439 | MH622575 | MH681376 | MH636497 | MH622707 |
| *C. siamense* | YN42-2-1 | Minzufengqingyuan, Chengdong, Xishuangbanna, Yunnan | Yaomang | leaf | MH622456 | MH622592 | MH681393 | MH636514 | MH622724 |
| *C. siamense* | YN47-2-1 | Mangguosenlingongyuan, Yuanjiang, Yunnan | Tainong | leaf | MH622457 | MH622593 | MH681394 | MH636515 | MH622725 |
| *C. siamense* | YN40-1-2 | Longmu, Nansha, Yuanyang, Yunnan | unknown | leaf | MH622441 | MH622577 | MH681378 | MH636499 | MH622709 |
| *C. fructicola* | GZ1-2 | Bawei, Zhexiang, Wangmo, Guizhou | Tainong | leaf | MH622365 | MH622501 | MH681306 | MH636427 | MH622637 |
| *C. fructicola* | GZ2-1 | Zhexiang, Wangmo, Guizhou | Tainong | leaf | MH622366 | MH622502 | MH681307 | MH636428 | MH622638 |
| *C. fructicola* | GZ4 | Zhexiang, Wangmo, Guizhou | Tainong | leaf | MH622367 | MH622503 | MH681308 | MH636429 | MH622639 |
| *C. fructicola* | GZ6-3 | Zhexiang, Wangmo, Guizhou | Tainong | leaf | MH622369 | MH622505 | MH681310 | MH636431 | MH622641 |
| *C. fructicola* | GZ8-3 | Zhexiang, Wangmo, Guizhou | Tainong | leaf | MH622370 | MH622506 | MH681311 | MH636432 | MH622642 |
| *C. fructicola* | GZ9 | Zhexiang, Wangmo, Guizhou | Tainong | leaf | MH622371 | MH622507 | MH681312 | MH636433 | MH622643 |
| *C. fructicola* | GZ13-1 | Qiaojiang, Youmai, Wangmo, Guizhou | Tainong | leaf | MH622375 | MH622511 | MH681315 | MH636436 | MH622646 |
| *C. fructicola* | GZ14-1 | Qiaojiang, Youmai, Wangmo, Guizhou | unknown | leaf | MH622376 | MH622512 | MH681316 | MH636437 | MH622647 |
| *C. fructicola* | GZ16 | Qiaojiang,Youmai, Wangmo, Guizhou | Tainong | leaf | MH622381 | MH622517 | MH681320 | MH636441 | MH622651 |
| *C. fructicola* | GZ15-1 | Qiaojiang, Youmai, Wangmo, Guizhou | Tainong | leaf | MH622380 | MH622516 | MH681319 | MH636440 | MH622650 |
| *C. fructicola* | GZ19-G-2 | Tianfang, Bajie, Xingyi, Guizhou | Tainong | Fruit | MH622383 | MH622519 | MH681321 | MH636442 | MH622652 |
| *C. fructicola* | GZ21-2 | TianZhai, Bajie, Xingyi, Guizhou | Tainong | leaf | MH622384 | MH622520 | MH681322 | MH636443 | MH622653 |
| *C. fructicola* | GZ12-1 | Wangmo city, Guizhou | Tainong | leaf | MH622374 | MH622510 | MH681314 | MH636435 | MH622645 |
| *C. fructicola* | GZ25-1 | Bannong, Yanjia, Ceheng, Guizhou | unknown | leaf | MH622387 | MH622523 | MH681324 | MH636445 | MH622655 |
| *C. fructicola* | HN7 | Nanxingnongchang, Yalongwan, Sanya, Hainan | Tainong | leaf | MH622388 | MH622524 | MH681325 | MH636446 | MH622656 |
| *C. fructicola* | HN19-1 | Nanbinnongchang, Yacheng, Sanya, Hainan | Tainong | leaf | MH622391 | MH622527 | MH681328 | MH636449 | MH622659 |
| *C. fructicola* | HN47-2 | Malong, Datian, Dongfang, Hainan | Tainong | leaf | MH622400 | MH622536 | MH681337 | MH636458 | MH622668 |
| *C. fructicola* | HN54-1 | Baoying, Tianya, Sanya, Hainan | Tainong | leaf | MH622402 | MH622538 | MH681339 | MH636460 | MH622670 |
| *C. fructicola* | YN13-1 | Longtou, Rongjiang, Huaping | Tainong | leaf | MH622432 | MH622568 | MH681369 | MH636490 | MH622700 |
| *C. fructicola* | YN21-1-3 | hongxinfenchang, Namaluosizhai, Ganzhuang, Yuanjiang, Yunnan | unknown | leaf | MH622443 | MH622579 | MH681380 | MH636501 | MH622711 |
| *C. fructicola* | YN30-4 | Ganba, Ganzhuang, Yuanjiang, Yunnan | unknown | leaf | MH622444 | MH622580 | MH681381 | MH636502 | MH622712 |
| *C. fructicola* | YN43-1 | Minzufengqingyuan, Chengdong, Xishuangbanna, Yunnan | Yaomang | leaf | MH622455 | MH622591 | MH681392 | MH636513 | MH622723 |
| *C. fructicola* | FJ13-3 | Miyuanshengtainongchang, Qiantign, Zhangpu, Fujian | Yuwen | leaf | MH622467 | MH622603 | MH681404 | MH636525 | MH622735 |
| *C. fructicola* | FJ17-5 | Miyuanshengtainongchang, Qiantign, Zhangpu, Fujian | Yuwen | leaf | MH622469 | MH622605 | MH681406 | MH636527 | MH622737 |
| *C. fructicola* | FJ25-1 | Jinling, Yanxi, Changtai, Fujian | Jinhuang | leaf | MH622471 | MH622607 | MH681408 | MH636529 | MH622739 |
| *C. fructicola* | FJ26-1 | Jinling, Yanxi, Changtai, Fujian | Jinhuang | leaf | MH622472 | MH622608 | MH681409 | MH636530 | MH622740 |
| *C. fructicola* | FJ27-2 | Jinling, Yanxi, Changtai, Fujian | Jinhuang | leaf | MH622473 | MH622609 | MH681410 | MH636531 | MH622741 |
| *C. fructicola* | FJ28-1 | Jinling, Yanxi, Changtai, Fujian | Guifei | leaf | MH622474 | MH622610 | MH681411 | MH636532 | MH622742 |
| *C. fructicola* | FJ29-1 | Jinling, Yanxi, Changtai, Fujian | Guifei | leaf | MH622475 | MH622611 | MH681412 | MH636533 | MH622743 |
| *C. fructicola* | FJ32-6 | Guanshan, Changtai, Zhangzhou, Fujian | unknown | leaf | MH622479 | MH622615 | MH681413 | MH636534 | MH622744 |
| *C. fructicola* | FJ34-5 | Zhelin, Longxin, Nanzhao, Pinghe, Fujian | unknown | leaf | MH622481 | MH622617 | MH681415 | MH636536 | MH622746 |
| *C. fructicola* | FJ35-5 | Xintian, Banzhai, Pinghe, Fujian | unknown | leaf | MH622483 | MH622619 | MH681417 | MH636538 | MH622748 |
| *C. karstii* | GZ5-2 | Zhexiang, Wangmo, Guizhou | Tainong | leaf | MH622368 | MH622504 | MH681309 | MH636430 | MH622640 |
| *C. karstii* | GZ14-2 | Qiaojiang, Youmai, Wangmo, Guizhou | unknown | leaf | MH622377 | MH622513 | MH681317 | MH636438 | MH622648 |
| *C. karstii* | HN20-1 | Nanshan, Sanya, Hainan | Tainong | leaf | MH622405 | MH622541 | MH681342 | MH636463 | MH622673 |
| *C. karstii* | YN34-1 | Lengdun, Baohe, Yuanyang, Yunnan | Xiangjiaomang | leaf | MH622448 | MH622584 | MH681385 | MH636506 | MH622716 |
| *C. karstii* | YN40-1-3 | Longmu, Nansha, Yuanyang, Yunnan | wild-unknown | leaf | MH622449 | MH622585 | MH681386 | MH636507 | MH622717 |
| *C. endophytica* | HN37-2 | #4 Century old Mango tree, Chahe, Changjiang, Hainan | wild-unkown | leaf | MH622394 | MH622530 | MH681331 | MH636452 | MH622662 |
| *C. endophytica* | HN37-6 | #4 Century old Mango tree, Chahe, Changjiang, Hainan | wild-unkown | leaf | MH622395 | MH622531 | MH681332 | MH636453 | MH622663 |
| *C. endophytica* | YN32-6 | Mangguosenlingongyuan, Yuanjiang, Yunnan | wild-unkown | leaf | MH622453 | MH622589 | MH681390 | MH636511 | MH622721 |
| *C. endophytica* | YN32-2 | Mangguosenlingongyuan, Yuanjiang, Yunnan | wild-unkown | leaf | MH622451 | MH622587 | MH681388 | MH636509 | MH622719 |
| *C. scovillei* | YN51-1 | Zhiwuyuan, Xishuangbanna, Yunnan | wild-unkown | leaf | MH622454 | MH622590 | MH681391 | MH636512 | MH622722 |
| *C. scovillei* | HN44-2 | Shuifu, Chahe, Changjiang, Hainan | Hongyu | leaf | MH622398 | MH622534 | MH681335 | MH636456 | MH622666 |
| *C. scovillei* | HN44-3 | Shuifu, Chahe, Changjiang, Hainan | Hongyu | leaf | MH622399 | MH622535 | MH681336 | MH636457 | MH622667 |
| *C. cliviicola* | YN8-2 | Wuxin, Xinzhuamg, Rongjiang, Huaping, Yunnan | Kate | leaf | MH622429 | MH622565 | MH681366 | MH636487 | MH622697 |
| *C. cliviicola* | YN31-4 | Ganba, Ganzhuang, Yuanjiang, Yunnan | Tainong | leaf | MH622450 | MH622586 | MH681387 | MH636508 | MH622718 |
| *C. gigasporum* | HN42-2 | #2 Century old Mango tree, Chahe, Changjiang, Hainan | wild-unkown | branch | MH622409 | MH622545 | MH681346 | MH636467 | MH622677 |
| *C. gloeosporioides* | GZ14-G-1 | Qiaojiang, Youmai, Wangmo, Guizhou | unkown | Fruit | MH622379 | MH622515 | MH681318 | MH636439 | MH622649 |
| *C. liaoningense* | YN33-1 | Hongtupo, Ganzhuang, Yuanjiang, Yunnan | unkown | leaf | MH622446 | MH622582 | MH681383 | MH636504 | MH622714 |
| *C. musae* | GZ23-3 | Bannong,Yanjia, Ceheng, Guizhou | Hongxiangya | leaf | MH622386 | MH622522 | MH681323 | MH636444 | MH622654 |
| *C. tropicale* | HN32-1 | Chigong, Jiusuo, ledong, Sanya, Hainan | Hongmang | leaf | MH622408 | MH622544 | MH681345 | MH636466 | MH622676 |
| *C. cordylinicola* | HN23-5 | Ducun, Yacheng, Sanya, Hainan | Jinhuang | leaf | MH622406 | MH622542 | MH681343 | MH636464 | MH622674 |

^1^ ACT: actin gene; CHS-1: chitin synthase; GAPDH: glyceraldehyde-3-phosphate dehydrogenase; ITS: rDNA internal transcribed spacer region; TUB2: β-tubulin2.

**Supplementary Table S2** Previously published sequences of *Colletotrichum* species and GenBank accession numbers used for phylogenetic analysis

| Species | Culture^1^ | Host | Country | GenBank accession numbers^2^ | | | | |
| --- | --- | --- | --- | --- | --- | --- | --- | --- |
|  |  |  |  | ACT | CHS-1 | GAPDH | ITS | TUB2 |
| *C. acutatum* | CBS 112996, ATCC 56816, STE-U 5292* | *Carica papaya* | Australia | JQ005839 | JQ005797 | JQ948677 | JQ005776 | JQ005860 |
| *C. acutatum* | IMI 384175, CPC 18936 | *Nerium oleander* | New Zealand | JQ949690 | JQ949030 | JQ948700 | JQ948369 | JQ950020 |
| *C. acutatum* | CBS 126521 | *Anemone hybride* | Netherlands | JQ949687 | JQ949027 | JQ948697 | JQ948366 | JQ950017 |
| *C. aenigma* | ICMP 18608* | *Pyrus pyrifolia* | Israel | JX009519 | JX009789 | JX009913 | JX010243 | JX010390 |
| *C. aeschynomenes* | ICMP 17673*, ATCC 201874 | *Aeschynomene virginica* | USA | JX009483 | JX009799 | JX009930 | JX010176 | JX010392 |
| *C. alienum* | ICMP 12071* | *Malus domestica* | New Zealand | JX009572 | JX009882 | JX010028 | JX010251 | JX010411 |
| *C. annellatum* | CBS 129826, CH1* | Hevea brasiliensis, leaf | Colombia | JQ005570 | JQ005396 | JQ005309 | JQ005222 | JQ005656 |
| *C. asianum* | IMI 313839 | *Mangifera indica* | Australia | JX009576 | JX009753 | JX009915 | JX010192 | JX010384 |
| *C. asianum* | HKUCC 10862, ICMP 18605 | *Mangifera indica* | Thailand | JX009465 | JX009787 | JX010021 | JX010194 | -- |
| *C. asianum* | MAFF 306627, ICMP 18603 | *Mangifera indica* | Philipines | JX009579 | JX009825 | JX009938 | JX010195 | -- |
| *C. asianum* | ICMP 18580 | *Coffea arabica* | Thailand | JX009584 | JX009867 | JX010053 | FJ972612 | JX010406 |
| *C. beeveri* | CBS 128527, ICMP 18594* | Brachyglottis repanda | New Zealand | JQ005519 | JQ005345 | JQ005258 | JQ005171 | JQ005605 |
| *C. boninense* | CBS 123755, MAFF 305972* | *Crinum asiaticum* var. *sinicum* | Japan | JQ005501 | JQ005327 | JQ005240 | JQ005153 | JQ005588 |
| *C. brasiliense* | CBS 128501, ICMP 18607, PAS12* | Passiflora edulis, fruit anthracnose | Brazil | JQ005583 | JQ005409 | JQ005322 | JQ005235 | JQ005669 |
| *C. brassicicola* | CBS 101059, LYN 16331* | Brassica oleracea var. gemmifera, leaf spot | New Zealand | JQ005520 | JQ005346 | JQ005259 | JQ005172 | JQ005606 |
| *C. brevisporum* | BCC 38876* | *Neoregalia* sp. | Thailand | JN050216 | KF687760 | JN050227 | JN050238 | JN050244 |
| *C. brevisporum* | COUFAL0053 | *Capsicum frutescens* | China | KY319123 | KY319129 | KY319099 | KY319117 | KY319108 |
| *C. brisbanense* | CBS 292.67, DPI 11711* | *Capsicum annuum* | Australia | JQ949612 | JQ948952 | JQ948621 | JQ948291 | JQ949942 |
| *C. chrysanthemi* | IMI 364540, CPC 18930 | *Chrysanthemum coronarium,* | China | JQ949594 | JQ948934 | JQ948603 | JQ948273 | JQ949924 |
| *C. clidemiae* | ICMP 18658* | *Clidemia hirta* | USA,Hawaii | JX009537 | JX009877 | JX009989 | JX010265 | JX010438 |
| *C. cliviea* | CBS 125375, CSSK4* | *Clivia miniata* | China | GQ856777 | GQ856722 | GQ856756 | GQ485607 | GQ849440 |
| *C. colombiense* | CBS 129818, G2* | Passiflora edulis, leaf | Colombia | JQ005522 | JQ005348 | JQ005261 | JQ005174 | JQ005608 |
| *C. constrictum* | CBS 128504, ICMP 12941* | Citrus limon, fruit rot | New Zealand | JQ005586 | JQ005412 | JQ005325 | JQ005238 | JQ005672 |
| *C. cordylinicola* | MFLUCC 090551*, ICMP 18579 | *Cordyline fruticosa* | Thailand | HM470235 | JX009864 | JX009975 | JX010226 | JX010440 |
| *C. costaricense* | CBS 330.75* | *Coffea arabica,* cv. *Typica* | Costa Rica | JQ949501 | JQ948841 | JQ948510 | JQ948180 | JQ949831 |
| *C. cuscutae* | IMI 304802, CPC 18873* | *Cuscuta* sp. | Dominica | JQ949516 | JQ948856 | JQ948525 | JQ948195 | JQ949846 |
| *C. cymbidiicola* | IMI 347923* | Cymbidium sp., leaf lesion | Australia | JQ005514 | JQ005340 | JQ005253 | JQ005166 | JQ005600 |
| *C. dacrycarpi* | CBS 130241, ICMP 19107* | Dacrycarpus dacrydioides, leaf endophyte | New Zealand | JQ005584 | JQ005410 | JQ005323 | JQ005236 | JQ005670 |
| *C. endophytica* | CAUG28 | *Capsicum annuum* | China | KP145329 | KP145385 | KP145413 | KP145441 | KP145469 |
| *C. fructicola* | ICMP 18727 | *Fragaria ananassa* | USA | JX009565 | JX009812 | JX010035 | JX010179 | JX010394 |
| *C. fructicola* | ICMP 18613 | *Limonium sinuatum* | Israel | JX009491 | JX009772 | JX009998 | JX010167 | JX010388 |
| *C. fructicola* (syn. *C. ignotum*) | CBS 125397 | *Tetragastris panamensis* | Panama | JX009581 | JX009874 | JX010032 | JX010173 | JX010409 |
| *C. fructicola* (syn. *Glomerella cingulata* var.*minor*) | CBS 238.49(*), ICMP 17921 | *Ficus edulis* | Germany | JX009495 | JX009839 | JX009923 | JX010181 | JX010400 |
| *C. gigasporum* | CBS 133266, MUCL 44947* | *Centella asiatica* | Madagascar |  | KF687761 | KF687822 | KF687715 | KF687866 |
| *C. gloeosporioides* | IMI 356878 | *Citrus sinensis* | Italy | JX009531 | JX009818 | JX010056 | JX010152 | JX010445 |
| *C. guajavae* | IMI 350839, CPC 18893* | *Psidium guajava* | India | JQ949591 | JQ948949 | JQ948618 | JQ948288 | JQ949921 |
| *C. hippeastri* | CBS 125376, CSSG1* | Hippeastrum vittatum, leaf | China | JQ005579 | JQ005405 | JQ005318 | JQ005231 | JQ005665 |
| *C. indonesiense* | CBS 127551, CPC 14986* | *Eucalyptus* sp. | Indonesia | JQ949609 | JQ948949 | JQ948618 | JQ948288 | JQ949939 |
| *C. jiangxiense* | LC3463, CGMCC 3.17363 | *Ca. sinensis* |  | KJ954471 | --- | KJ954902 | KJ955201 | KJ955348 |
| *C. kahawae* subsp. *ciggaro* | ICMP 18539* | *Olea europaea* | Australia | JX009523 | JX009800 | JX009966 | JX010230 | JX010434 |
| *C. kahawae* subsp. *ciggaro* | CBS 112984, ICMP 17932 | *Dryandra* sp. | South Africa | JX009434 | JX009833 | JX009973 | JX010237 | - |
| *C. kahawae* subsp. *ciggaro (syn. Glomerella* | CBS 237.49(*), ICMP 17922 | *Hypericum perforatum* | Germany | JX009450 | JX009840 | JX010042 | JX010238 | JX010432 |
| *C. kahawae* subsp. *kahawae* | CBS 982.69, ICMP 17915 | *Coffea arabica* | Angola | JX009474 | JX009829 | JX010040 | JX010234 | JX010435 |
| *C. karstii* | CBS 128500, ICMP 18585 | *Annona cherimola* | New Zealand | JQ005550 | JQ005376 | JQ005289 | JQ005202 | JQ005636 |
| *C. karstii* | CBS 118401 | *Pachira* sp. | China | JQ005540 | JQ005279 | JQ005366 | JQ005192 | JQ005626 |
| *C. karstii* | CBS 128524, ICMP 18588 | *Citrullus lanatus* | New Zealand | JQ005543 | JQ005369 | JQ005282 | JQ005195 | JQ005629 |
| *C. karstii* | CBS 132134, CGMCC3.14194*. | Vandasp. | China | HM581995 | HM582023 | HM585391 | HM585409 | HM585428 |
| *C. laticiphilum* | CBS 112989, IMI 383015, STE-U 5303 | *Hevea brasiliensis* | India | JQ949610 | JQ948950 | JQ948619 | JQ948289 | JQ949940 |
| *C. liaoningense* | CAUOS2 | *Capsicum* sp. | China | KP890097 | KP890127 | KP890135 | KP890104 | KP890111 |
| *C. lupini* | CBS 109225, BBA 70884* | *Lupinus albus* | Ukraine | JQ949476 | JQ948816 | JQ948485 | JQ948155 | JQ949806 |
| *C. musae* | CBS 116870*, ICMP 19119 | *Musa* sp. | USA | JX009433 | JX009896 | JX010050 | JX010146 | HQ596280 |
| *C. novae-zelandiae* | CBS 128505, ICMP 12944* | Capsicum annuum, fruit rot | New Zealand | JQ005576 | JQ005402 | JQ005315 | JQ005228 | JQ005662 |
| *C. nupharicola* | CBS 469.96*, ICMP 17938 | *Nuphar lutea* subsp*. Polysepala* | USA | JX009486 | JX009834 | JX009936 | JX010189 | JX010397 |
| *C. nymphaeae* | MEP 1534, CBS 119294/BPI 871836 | *Leucaena* sp. | Mexico | JQ949526 | JQ948866 | JQ948535 | JQ948205 | JQ949856 |
| *C. nymphaeae* | CBS 129926, CPC 18719 | Litter | Thailand | JQ949537 | JQ948877 | JQ948546 | JQ948216 | JQ949867 |
| *C. nymphaeae* | IMI 311743 | *Fragaria × ananassa* | USA | JQ949579 | JQ948919 | JQ948588 | JQ948258 | JQ949909 |
| *C. nymphaeae* | IMI 345032 | *Fragaria × ananassa* | Italy | JQ949562 | JQ948902 | JQ948571 | JQ948241 | JQ949892 |
| *C. nymphaeae* | CBS515.78* | *Nymphaea alba* | Netherlands | JQ949518 | JQ948858 | JQ948527 | JQ948197 | JQ949848 |
| *C. oncidii* | CBS 129828* | Oncidium sp., leaf | Germany | JQ005517 | JQ005343 | JQ005256 | JQ005169 | JQ005603 |
| *C. parsonsiae* | CBS 128525, ICMP 18590* | Parsonsia capsularis, leaf endophyte | New Zealand | JQ005581 | JQ005407 | JQ005320 | JQ005233 | JQ005667 |
| *C. paxtonii* | CBS 502.97, LARS 58 | *Musa nana* | “West Indies” | JQ949607 | JQ948947 | JQ948616 | JQ948286 | JQ949937 |
| *C. queenslandicum* | ICMP 1778* | *Carica papaya* | Australia | JX009447 | JX009899 | JX009934 | JX010276 | JX010414 |
| *C. scovillei* | CAUA1 | *Capsicum annuum* | China | KP145112 | KP145272 | KP145144 | KP145208 | KP145240 |
| *C. scovillei* | CBS 126529, PD 94/921-3, BBA 70349* | *Capsicum* sp. | Indonesia | JQ949588 | JQ948928 | JQ948597 | JQ948267 | JQ949918 |
| *C. scovillei* | CBS 120708, HKUCC 10893, Mj6 | *Capsicum annuum* | Thailand | JQ949590 | JQ948930 | JQ948599 | JQ948269 | JQ949920 |
| *C. siamense* | ICMP 17795 | *Malus domestica* | USA | JX009506 | JX009805 | JX010051 | JX010162 | JX010393 |
| *C. siamense* | ICMP 18578 | *Coffea arabica* | Thailand | FJ907423 | JX009865 | JX009924 | JX010171 | JX010404 |
| *C. siamense* | DAR76934, ICMP 18574 | *Pistacia vera* | Australia | JX009535 | JX009798 | JX010002 | JX010270 | JX010391 |
| *C. siamense* | CBS 113199, STE-U 2290, JT 813 | *Protea cynaroides* | Zimbabwe | KC296930 | KC296985 | KC297008 | KC297066 | KC297090 |
| *C. siamense* (syn. *C. hymenocallidis*) | CBS 125378(*), ICMP 18642 | *Hymenocallis americana* | China | GQ856775 | GQ856730 | JX010019 | JX010278 | JX010410 |
| *C. simmondsii* | IMI 313840, CPC 18875 | *Mangifera indica* | Australia | JQ949605 | JQ948945 | JQ948614 | JQ948284 | JQ949935 |
| *C. sloanei* | IMI 364297, CPC 18929* | *Theobroma cacao* | Malaysia | JQ949608 | JQ948948 | JQ948617 | JQ948287 | JQ949938 |
| *C. tamarilloi* | CBS 129814, T.A.6* | *Solanum betaceum* | Colombia | JQ949505 | JQ948845 | JQ948514 | JQ948184 | JQ949835 |
| *C. theobromicola* | CBS 124945*, ICMP 18649 | *Theobroma cacao* | Panama | JX009444 | JX009869 | JX010006 | JX010294 | JX010447 |
| *C. torulosum* | CBS 128544, ICMP 18586* | Solanum melongena | New Zealand | JQ005512 | JQ005338 | JQ005251 | JQ005164 | JQ005598 |
| *C. tropicale* | CBS 124949*, ICMP 18653 | *Theobroma cacao* | Panama | JX009489 | JX009870 | JX010007 | JX010264 | JX010407 |
| *C. walleri* | CBS 125472, BMT(HL)19* | *Coffea* sp. | Vietnam | JQ949596 | JQ948936 | JQ948605 | JQ948275 | JQ949926 |
| *C. xanthorrhoeae* | BRIP 45094*, ICMP 17903, CBS 127831 | *Xanthorrhoea preissii* | Australia | JX009478 | JX009823 | JX009927 | JX010261 | JX010448 |
| *Glomerella cingulata* “f.sp. *camelliae*” | ICMP 18542, LF899 | *Camellia sasanqua* | USA | JX009488 | JX009857 | JX009994 | JX010223 | JX010429 |
| *Monilochaetes infuscans* | CBS 869.96* | *Ipomoea batatas* | South Africa | JQ005843 | JQ005801 | JX546612 | JQ005780 | JQ005864 |

^1^ IMI: Culture collection of CABI Europe UK Centre, Egham, UK; ICMP: International Collection of Microorganisms from Plant, Landcare Research, Auckland, New Zealand; CBS: CBS-KNAW, Fungal Biodiversity Centre, Utrecht, The Netherlands; UASB: BRIP: Plant Pathology Herbarium, Department of Employment, Economic, Development and Innovation, Queensland, Australia. *ex-holotype or ex-epitype cultures.

^2^ ACT: actin gene; CHS-1: chitin synthase; GAPDH: glyceraldehyde-3-phosphate dehydrogenase; ITS: rDNA internal transcribed spacer region; TUB2: β-tubin2.

**Supplementary Table S3** Mycelial growth, conidial size and aggressiveness descriptions for *Colletotrichum* isolates on mango (*Mangifera indica* L.) in China

| Species | Isolates | Mycelial growth (Mean±SD, cm/d) ^1^ | Lesion diameter on fruit (Mean±SD, cm) ^2^ | Lesion diameter on Leaf (Mean±SD, cm) ^3^ | Conidial size (Length±SD×Width±SD, μm) ^4^ |
| --- | --- | --- | --- | --- | --- |
| *C. asianum* | GD4-1 | 1.05±0.05 | 0.67±0.05 | 2.34±0.08 | 17.10±0.12×5.31±0.04 |
| *C. asianum* | GD16 | 1.05±0.10 | 1.53±0.12 | 1.30±0.05 | 15.06±0.09×4.97±0.03 |
| *C. asianum* | GD27 | 1.59±0.04 | 0.96±0.11 | 2.17±0.08 | 15.70±0.14×5.96±0.40 |
| *C. asianum* | HN29-4 | 1.01±0.02 | 0.79±0.02 | 1.59±0.06 | 16.57±0.12×4.76±0.03 |
| *C. asianum* | HN34-1 | 1.32±0.01 | 1.19±0.08 | 1.95±0.04 | 16.72±0.16×5.11±0.37 |
| *C. asianum* | SC4-1 | 1.10±0.01 | 0.70±0.03 | 2.38±0.15 | 17.10±0.12×5.19±0.04 |
| *C. asianum* | SC19 | 1.21±0.01 | 1.31±0.13 | 1.81±0.06 | 13.66±0.13×5.31±0.04 |
| *C. asianum* | SC39 | 1.25±0.02 | 1.65±0.08 | 1.93±0.07 | 15.09±0.13×5.17±0.04 |
| *C. asianum* | YN2-1 | 1.17±0.02 | 0.66±0.03 | 1.44±0.04 | 14.56±0.19×5.95±0.08 |
| *C. asianum* | YN3-1 | 1.23±0.03 | 1.56±0.15 | 1.20±0.05 | 12.98±0.13×6.39±0.05 |
| *C. asianum* | YN4-1 | 1.16±0.01 | 1.57±0.09 | 1.69±0.06 | 13.21±0.12×5.14±0.48 |
| *C. asianum* | YN6-1-1 | 0.83±0.06 | 2.01±0.10 | 1.60±0.07 | 15.49±0.13×5.15±0.05 |
| *C. asianum* | YN7-1 | 1.09±0.02 | 0.68±0.03 | 1.88±0.07 | 18.62±0.14×5.67±0.04 |
| *C. asianum* | YN11-2-1 | 1.18±0.02 | 1.08±0.06 | 1.65±0.06 | 13.02±0.12×5.61±0.04 |
| *C. asianum* | YN12-2-1 | 1.26±0.02 | 1.24±0.14 | 1.61±0.07 | 17.25±0.16×5.32±0.06 |
| *C. asianum* | YN4-2-1 | 1.02±0.01 | 1.53±0.17 | 1.50±0.07 | 16.26±0.11×5.65±0.06 |
| *C. asianum* | YN18 | 1.18±0.02 | 2.29±0.09 | 1.37±0.05 | 12.95±0.10×4.97±0.05 |
| *C. asianum* | YN19-1-1 | 1.09±0.06 | 1.20±0.10 | 1.42±0.07 | 14.59±0.09×6.08±0.04 |
| *C. asianum* | YN27-2-3 | 0.87±0.04 | 1.19±0.08 | 1.45±0.06 | 16.61±0.12×4.92±0.03 |
| *C. asianum* | YN56-2-1 | 1.09±0.09 | 0.64±0.03 | 1.07±0.06 | 18.28±0.19×5.38±0.03 |
| *C. asianum* | YN25-1-2 | 1.03±0.08 | 1.37±0.16 | 1.30±0.07 | 17.60±0.20×4.96±0.09 |
| *C. asianum* | YN55-1 | 0.90±0.09 | 0.96±0.19 | 0.89±0.08 | 14.50±0.17×5.53±0.04 |
| *C. asianum* | YN29-1 | 0.90±0.05 | 0.85±0.07 | 1.25±0.08 | 16.46±0.14×6.18±0.06 |
| *C. asianum* | YN27-1-1 | 0.38±0.05 | 0.76±0.03 | 1.38±0.08 | 18.01±0.12×5.34±0.03 |
| *C. asianum* | FJ1-3 | 1.06±0.02 | 0.90±0.07 | 1.47±0.09 | 16.77±0.16×5.20±0.06 |
| *C. asianum* | FJ4-2 | 0.98±0.02 | 1.01±0.02 | 1.04±0.05 | 11.94±0.23×5.08±0.09 |
| *C. asianum* | FJ5-1 | 0.99±0.05 | 3.21±1.40 | 1.54±0.12 | 12.28±0.16×4.56±0.09 |
| *C. asianum* | FJ6-4 | 1.10±0.15 | 1.46±0.11 | 1.05±0.04 | 17.51±0.12×5.38±0.07 |
| *C. asianum* | FJ7-3 | 0.97±0.05 | 1.39±0.09 | 1.52±0.03 | 17.98±0.19×5.02±0.05 |
| *C. asianum* | FJ9-1 | 1.10±0.03 | 1.43±0.09 | 1.83±0.05 | 15.81±0.01×5.64±0.06 |
| *C. asianum* | FJ10-4 | 1.00±0.04 | 1.44±0.06 | 1.67±0.06 | 16.19±0.16×5.47±0.06 |
| *C. asianum* | FJ11-1 | 0.95±0.02 | 1.03±0.04 | 3.36±1.46 | 16.92±0.15×5.13±0.06 |
| *C. asianum* | FJ12-4 | 0.74±0.06 | 1.07±0.02 | 1.98±0.06 | 17.58±0.16×5.74±0.06 |
| *C. asianum* | FJ15-1 | 1.13±0.02 | 1.02±0.04 | 1.73±0.10 | 14.36±0.15×5.33±0.09 |
| *C. asianum* | FJ18-3 | 0.90±0.07 | 0.88±0.04 | 1.47±0.04 | 20.27±0.28×5.28±0.04 |
| *C. asianum* | FJ33-2 | 1.05±0.07 | 1.61±0.10 | 1.39±0.04 | 16.44±0.13×5.47±0.03 |
| *C. asianum* | FJ35-1 | 1.12±0.04 | 1.48±0.14 | 1.77±0.04 | 16.70±0.15×5.67±0.04 |
| *C. asianum* | FJ36-6 | 1.04±0.03 | 0.98±0.10 | 1.47±0.05 | 18.72±0.22×5.38±0.03 |
| *C. siamense* | GD6-2 | 0.66±0.04 | 1.44±0.10 | 1.96±0.04 | 14.13±0.10×5.28±0.03 |
| *C. siamense* | GD7-1 | 1.05±0.02 | 3.64±0.39 | 2.72±0.13 | 14.42±0.10×4.98±0.03 |
| *C. siamense* | GD8-1 | 1.00±0.01 | 2.25±0.19 | 2.77±0.07 | 14.70±0.13×5.65±0.03 |
| *C. siamense* | GD10-1 | 0.94±0.05 | 3.37±0.20 | 3.69±0.91 | 14.31±0.11×5.24±0.04 |
| *C. siamense* | GD11-1 | 0.94±0.09 | 0.85±0.03 | 1.78±0.06 | 14.46±0.11×5.51±0.04 |
| *C. siamense* | GD12-1 | 0.62±0.08 | 0.92±0.06 | 1.30±0.04 | 12.71±0.15×3.96±0.05 |
| *C. siamense* | GD13-1 | 0.88±0.02 | 0.70±0.04 | 1.96±0.05 | 12.63±0.15×5.48±0.08 |
| *C. siamense* | GD14-2 | 1.25±0.07 | 1.29±0.05 | 1.78±0.11 | 14.51±0.10×5.21±0.06 |
| *C. siamense* | GD17 | 0.91±0.03 | 1.14±0.13 | 3.14±0.13 | 14.93±0.11×5.24±0.03 |
| *C. siamense* | GD29-1 | 1.51±0.06 | 1.74±0.08 | 1.90±0.06 | 12.29±0.08×5.04±0.04 |
| *C. siamense* | GD29-2 | 0.98±0.01 | 1.03±0.07 | 2.37±0.08 | 16.14±0.13×4.95±0.03 |
| *C. siamense* | GD30-2 | 1.16±0.02 | 1.56±0.17 | 2.36±0.09 | 16.24±0.16×5.39±0.04 |
| *C. siamense* | GD30-1 | 1.01±0.01 | 0.96±0.04 | 2.30±0.15 | 13.75±0.08×5.22±0.03 |
| *C. siamense* | HN10 | 0.63±0.01 | 0.78±0.08 | 2.45±0.06 | 16.32±0.13×5.15±0.04 |
| *C. siamense* | HN18-1 | 0.86±0.09 | 1.43±0.12 | 2.29±0.09 | 16.62±0.11×5.39±0.03 |
| *C. siamense* | HN29-2 | 0.97±0.04 | 1.45±0.11 | 0.76±0.06 | 15.24±0.09×5.57±0.04 |
| *C. siamense* | HN39-2 | 0.84±0.12 | 0.65±0.03 | 2.75±0.05 | 15.28±0.11×5.46±0.05 |
| *C. siamense* | HN39-3 | 0.58±0.03 | 1.57±0.09 | 2.72±0.10 | 15.28±0.10×5.18±0.04 |
| *C. siamense* | HN50-2 | 0.52±0.01 | 1.88±0.12 | 2.52±0.08 | 17.59±0.17×5.20±0.04 |
| *C. siamense* | HN58-1 | 1.29±0.03 | 2.01±0.13 | 2.33±0.08 | 14.72±0.10×5.33±0.05 |
| *C. siamense* | HN8-1 | 0.91±0.04 | 0.92±0.06 | 2.35±0.07 | 14.71±0.12×4.91±0.03 |
| *C. siamense* | HN22-3 | 0.92±0.01 | 0.86±0.07 | 2.12±0.07 | 12.57±0.11×5.34±0.06 |
| *C. siamense* | SC2-1 | 1.17±0.01 | 0.78±0.03 | 4.34±1.52 | 15.09±0.13×4.41±0.03 |
| *C. siamense* | SC3-1 | 1.01±0.03 | 0.87±0.10 | 1.84±0.07 | 13.97±0.12×5.20±0.04 |
| *C. siamense* | SC3-2 | 1.09±0.03 | 1.66±0.22 | 2.92±0.12 | 16.10±0.12×4.89±0.04 |
| *C. siamense* | SC4-3 | 0.99±0.01 | 1.88±0.16 | 3.02±0.08 | 16.21±0.17×5.26±0.03 |
| *C. siamense* | SC5 | 1.15±0.02 | 2.07±0.14 | 1.44±0.06 | 13.61±0.10×4.62±0.04 |
| *C. siamense* | SC16 | 0.97±0.08 | 0.54±0.01 | 2.19±0.06 | 22.79±0.28×5.37±0.04 |
| *C. siamense* | SC37-2 | 1.03±0.02 | 1.49±0.08 | 1.51±0.08 | 15.36±0.10×5.62±0.03 |
| *C. siamense* | SC38-1-1 | 1.19±0.03 | 0.89±0.05 | 1.83±0.10 | 14.19±0.10×5.46±0.04 |
| *C. siamense* | SC38-1-2 | 1.10±0.01 | 1.67±0.11 | 1.46±0.07 | 12.56±0.10×4.89±0.03 |
| *C. siamense* | SC38-1-3 | 1.00±0.01 | 1.37±0.09 | 2.42±0.06 | 14.82±0.16×4.81±0.04 |
| *C. siamense* | YN45-2-1 | 0.66±0.10 | 0.74±0.06 | 1.84±0.05 | 13.85±0.27×3.72±0.08 |
| *C. siamense* | YN28-1 | 0.90±0.13 | 0.76±0.05 | 2.10±0.05 | 13.16±0.11×6.07±0.07 |
| *C. siamense* | YN56-1-1 | 1.08±0.03 | 0.85±0.09 | 0.60±0.03 | 15.50±0.09×5.22±0.04 |
| *C. siamense* | YN42-2-1 | 0.82±0.11 | 0.83±0.04 | 1.60±0.08 | 15.78±0.10×5.09±0.03 |
| *C. siamense* | YN47-2-1 | 1.02±0.04 | 0.74±0.11 | 1.65±0.10 | 16.00±0.11×4.93±0.03 |
| *C. siamense* | YN40-1-2 | 0.91±0.05 | 1.03±0.12 | 1.21±0.06 | 14.90±0.11×5.57±0.04 |
| *C. fructicola* | GZ1-2 | 1.08±0.01 | 1.00±0.08 | 1.56±0.07 | 14.85±0.13×5.08±0.04 |
| *C. fructicola* | GZ2-1 | 1.08±0.01 | 0.85±0.05 | 1.65±0.12 | 13.97±0.13×5.90±0.07 |
| *C. fructicola* | GZ4 | 1.19±0.02 | 1.40±0.08 | 1.88±0.10 | 13.83±0.12×5.31±0.05 |
| *C. fructicola* | GZ6-3 | 0.86±0.01 | 0.77±0.07 | 1.40±0.08 | 13.35±0.08×6.90±0.04 |
| *C. fructicola* | GZ8-3 | 1.06±0.01 | 0.75±0.06 | 1.83±0.10 | 13.25±0.13×6.12±0.04 |
| *C. fructicola* | GZ9 | 1.00±0.02 | 0.87±0.08 | 1.76±0.09 | 13.15±0.07×5.94±0.04 |
| *C. fructicola* | GZ13-1 | 1.02±0.01 | 1.22±0.10 | 1.81±0.04 | 12.16±0.11×5.05±0.05 |
| *C. fructicola* | GZ14-1 | 1.06±0.03 | 1.28±0.07 | 1.66±0.09 | 13.17±0.11×5.44±0.04 |
| *C. fructicola* | GZ16 | 1.11±0.03 | 1.14±0.11 | 1.63±0.06 | 13.71±0.11×6.65±0.08 |
| *C. fructicola* | GZ15-1 | 1.07±0.02 | 0.80±0.05 | 1.23±0.09 | 14.97±0.09×6.98±0.03 |
| *C. fructicola* | GZ19-G-2 | 0.97±0.09 | 0.62±0.03 | 1.72±0.05 | 15.59±0.12×5.68±0.06 |
| *C. fructicola* | GZ21-2 | 1.11±0.02 | 1.29±0.07 | 2.06±0.06 | 12.90±0.12×6.10±0.06 |
| *C. fructicola* | GZ12-1 | 1.03±0.03 | 0.70±0.05 | 1.57±0.08 | 12.77±0.19×5.91±0.07 |
| *C. fructicola* | GZ25-1 | 0.98±0.08 | 0.74±0.04 | 1.44±0.10 | 11.51±0.08×6.05±0.05 |
| *C. fructicola* | HN7 | 0.90±0.04 | 1.08±0.05 | 2.14±0.10 | 13.65±0.24×6.36±0.10 |
| *C. fructicola* | HN19-1 | 1.03±0.02 | 1.32±0.13 | 2.11±0.07 | 15.03±0.11×5.02±0.04 |
| *C. fructicola* | HN47-2 | 0.68±0.03 | 1.22±0.03 | 2.44±0.11 | 14.50±0.10×5.20±0.03 |
| *C. fructicola* | HN54-1 | 1.03±0.03 | 1.65±0.15 | 1.80±0.10 | 12.03±0.22×5.31±0.06 |
| *C. fructicola* | YN13-1 | 0.96±0.01 | 1.03±0.06 | 2.10±0.06 | 11.93±0.20×5.84±0.07 |
| *C. fructicola* | YN21-1-3 | 1.06±0.03 | 1.95±0.18 | 1.71±0.07 | 13.46±0.14×6.42±0.05 |
| *C. fructicola* | YN30-4 | 0.89±0.07 | 0.75±0.07 | 1.77±0.07 | 15.82±0.12×5.11±0.03 |
| *C. fructicola* | YN43-1 | 0.84±0.10 | 1.03±0.11 | 1.39±0.07 | 16.80±0.12×5.42±0.03 |
| *C. fructicola* | FJ13-3 | 0.62±0.00 | 1.42±0.07 | 2.03±0.11 | 16.02±0.14×5.39±0.05 |
| *C. fructicola* | FJ17-5 | 0.62±0.12 | 0.71±0.02 | 1.85±0.08 | 14.04±0.15×5.42±0.04 |
| *C. fructicola* | FJ25-1 | 1.00±0.03 | 0.74±0.04 | 1.51±0.10 | 15.25±0.09×5.23±0.03 |
| *C. fructicola* | FJ26-1 | 0.60±0.03 | 1.10±0.06 | 1.55±0.09 | 14.92±0.10×5.18±0.04 |
| *C. fructicola* | FJ27-2 | 1.01±0.04 | 1.29±0.06 | 1.61±0.06 | 13.44±0.10×5.62±0.04 |
| *C. fructicola* | FJ28-1 | 0.94±0.03 | 1.39±0.10 | 2.02±0.08 | 15.92±0.13×4.72±0.04 |
| *C. fructicola* | FJ29-1 | 0.98±0.31 | 0.74±0.04 | 2.15±0.08 | 15.01±0.11×5.53±0.04 |
| *C. fructicola* | FJ32-6 | 1.07±0.04 | 1.11±0.09 | 1.96±0.09 | 14.95±0.11×5.66±0.03 |
| *C. fructicola* | FJ34-5 | 1.05±0.01 | 1.05±0.10 | 1.75±0.04 | 15.19±0.14×6.00±0.04 |
| *C. fructicola* | FJ35-5 | 0.98±0.03 | 1.32±0.11 | 2.18±0.04 | 13.34±0.07×5.82±0.03 |
| *C. karstii* | GZ5-2 | 1.04±0.01 | 1.11±0.06 | 2.23±0.08 | 13.50±0.10×6.08±0.05 |
| *C. karstii* | GZ14-2 | 1.03±0.01 | 1.18±0.07 | 1.71±0.09 | 14.32±0.08×6.60±0.05 |
| *C. karstii* | HN20-1 | 0.99±0.06 | 0.56±0.01 | 1.17±0.08 | 13.31±0.10×6.06±0.04 |
| *C. karstii* | YN34-1 | 0.90±0.02 | 0.67±0.02 | 1.31±0.09 | 15.07±0.12×6.74±0.05 |
| *C. karstii* | YN40-1-3 | 0.88±0.02 | 0.61±0.03 | 0.62±0.03 | 9.43±0.21×3.35±0.08 |
| *C. endophytica* | HN37-2 | 0.54±0.03 | 2.23±0.16 | 2.66±0.06 | 15.67±0.10×5.70±0.04 |
| *C. endophytica* | HN37-6 | 1.08±0.35 | 1.05±0.12 | 2.77±0.05 | 14.34±0.11×5.62±0.03 |
| *C. endophytica* | YN32-6 | 0.99±0.08 | 1.52±0.17 | 1.30±0.06 | 15.77±0.10×5.56±0.07 |
| *C. endophytica* | YN32-2 | 0.92±0.11 | 1.74±0.15 | 1.78±0.10 | 15.54±0.09×5.59±0.03 |
| *C. scovillei* | YN51-1 | 0.91±0.00 | 0.71±0.04 | 0.70±0.03 | 12.60±0.18×5.25±0.04 |
| *C. scovillei* | HN44-2 | 0.89±0.02 | 0.85±0.06 | 1.18±0.05 | 12.31±0.11×4.12±0.03 |
| *C. scovillei* | HN44-3 | 0.90±0.03 | 0.74±0.06 | 0.96±0.05 | 11.79±0.11×5.15±0.04 |
| *C. cliviicola* | YN8-2 | 1.24±0.01 | 0.62±0.04 | 1.28±0.13 | 18.32±0.14×5.99±0.04 |
| *C. cliviicola* | YN31-4 | 1.03±0.05 | 0.70±0.03 | 1.44±0.10 | 15.34±0.10×5.98±0.03 |
| *C. gigasporum* | HN42-2 | 0.93±0.04 | 0.66±0.02 | 1.92±0.06 | 25.54±0.55×8.22±0.15 |
| *C. gloeosporioides* | GZ14-G-1 | 1.03±0.02 | 1.44±0.13 | 1.81±0.05 | 15.49±0.16×5.22±0.03 |
| *C. liaoningense* | YN33-1 | 0.95±0.06 | 0.61±0.02 | 1.45±0.07 | 22.32±0.14×4.74±0.23 |
| *C. musae* | GZ23-3 | 0.77±0.02 | 1.06±0.17 | 1.35±0.09 | 13.45±0.13×4.13±0.26 |
| *C. tropicale* | HN32-1 | 1.12±0.02 | 1.98±0.20 | 2.34±0.05 | 14.1±0.09×5.29±0.02 |
| *C. cordylinicola* | HN23-5 | 0.73±0.14 | 0.61±0.02 | 1.28±0.04 | 10.84±0.13×5.63±0.05 |

**^1^** The colony diameter (cm) was measured in two perpendicular directions after 3, 5, 7 and 10 days on potato dextrose agar. The colony diameter data were used to calculate the mycelial growth rate (cm/day).

**^2, 3^** The aggressiveness of the isolates was evaluated by measuring lesion length at 7 days post inoculation (DPI) and 10 DPI in two perpendicular directions on leaves and fruits, respectively.

**^4^** The length and width of 120 conidia per isolate on PDA were measured after three weeks at 25°C.

**Supplementary Table S4** Primers used for PCR amplification and DNA sequencing

| Gene ^1^ | Primer | Primer sequences | References |
| --- | --- | --- | --- |
| ACT | ACT-512F | ATGTGCAAGGCCGGTTTCGC | Carbone^1^et al., 1999 |
|  | ACT-783R | TACGAGTCCTTCTGGCCCAT |  |
| GAPDH | GDF1 | GCCGTCAACGACCCCTTCATTGA | Guerber^2^ et al., 2003 |
|  | GDR1 | GGGTGGAGTCGTACTTGAGCATGT | Templeton^3^ et al, 1992 |
| CHS-1 | CHS-1-79F | TGGGGCAAGGATGCTTGGAAGAAG | Carbone^1^ et al., 1999 |
|  | CHS-1-354R | TGGAAGAACCATCTGTGAGAGTTG |  |
| TUB2 | T1 | AACATGCGTGAGATTGTAAGT | O’Donnell^4^ et a., 2000 |
|  | βt2b | ACCCTCAGTGTAGTGACCCTTGGC | Glass and Donaldson^5^, 1995 |
| ITS | ITS1 | TCCGTAGGTGAACCTGCGG | White^6^ et al., 1990 |
|  | ITS4 | TCCTCCGCTTATTGATATGC |  |

^1^ ACT: actin gene; GAPDH: glyceraldehyde-3-phosphate dehydrogenase; CHS-1: chitin synthase; TUB2: β-tubin2; ITS: rDNA internal transcribed spacer region.

**References**

1. Carbone, I. & Kohn, L. A method for designing primer sets for speciation studies in filamentous ascomycetes. *Mycologia* 91: 553-556 (1999).
2. Guerber, J. C., Liu, B., Correll, J. C., & Johnston, P. R. Characterization of diversity in *Colletotrichum acutatum* sensu lato by sequence analysis of two gene introns, mtDNA and intron RFLPs, and mating compatibility. *Mycologia*, 95: 872-895 (2003).
3. Templeton, M. D., Rikkerink, E. H., Solon, S. L. & Crowhurst, R. N. Cloning and molecular characterization of the glyceraldehyde-3-phosphate dehydrogenase-encoding gene and cDNA from the plant pathogenic fungus *Glomerella cingulata*. *Gene* 122: 225-230 (1992).
4. O’Donnell, K., Nirenberg, H. I., Aoki, T. & Cigelnik, E. A multigene phylogeny of the *Gibberella fujikuroi* species complex: detection of additional phylogenetically distinct species. *Mycoscience* 41: 61-78 (2000).
5. Glass, N. L. & Donaldson, G. C. Development of primer sets designed for use with the PCR to amplify conserved genes from filamentous ascomycetes. *Appl. Environ. Microbiol*. 61:1323-1330 (1995).
6. White, T. J., Bruns, T., Lee, S. J. W. T. & Taylor, J. W. Amplification and direct sequencing of fungal ribosomal RNA genes for phylogenetics. PCR protocols: a guide to methods and applications. 18: 315-322 (1990).
